# Magnetic resonance imaging of the dopamine system in schizophrenia – A scoping review

**DOI:** 10.3389/fpsyt.2022.925476

**Published:** 2022-09-20

**Authors:** Julia Schulz, Juliana Zimmermann, Christian Sorg, Aurore Menegaux, Felix Brandl

**Affiliations:** ^1^Department of Neuroradiology, School of Medicine, Technical University of Munich, Munich, Germany; ^2^TUM-NIC Neuroimaging Center, School of Medicine, Technical University of Munich, Munich, Germany; ^3^Department of Psychiatry and Psychotherapy, School of Medicine, Technical University of Munich, Munich, Germany

**Keywords:** dopamine, schizophrenia, magnetic resonance imaging, substantia nigra, ventral tegmental area

## Abstract

For decades, aberrant dopamine transmission has been proposed to play a central role in schizophrenia pathophysiology. These theories are supported by human *in vivo* molecular imaging studies of dopamine transmission, particularly positron emission tomography. However, there are several downsides to such approaches, for example limited spatial resolution or restriction of the measurement to synaptic processes of dopaminergic neurons. To overcome these limitations and to measure complementary aspects of dopamine transmission, magnetic resonance imaging (MRI)-based approaches investigating the macrostructure, metabolism, and connectivity of dopaminergic nuclei, i.e., substantia nigra pars compacta and ventral tegmental area, can be employed. In this scoping review, we focus on four dopamine MRI methods that have been employed in patients with schizophrenia so far: neuromelanin MRI, which is thought to measure long-term dopamine function in dopaminergic nuclei; morphometric MRI, which is assumed to measure the volume of dopaminergic nuclei; diffusion MRI, which is assumed to measure fiber-based structural connectivity of dopaminergic nuclei; and resting-state blood-oxygenation-level-dependent functional MRI, which is thought to measure functional connectivity of dopaminergic nuclei based on correlated blood oxygenation fluctuations. For each method, we describe the underlying signal, outcome measures, and downsides. We present the current state of research in schizophrenia and compare it to other disorders with either similar (psychotic) symptoms, i.e., bipolar disorder and major depressive disorder, or dopaminergic abnormalities, i.e., substance use disorder and Parkinson’s disease. Finally, we discuss overarching issues and outline future research questions.

## Introduction

**FIGURE 1:**
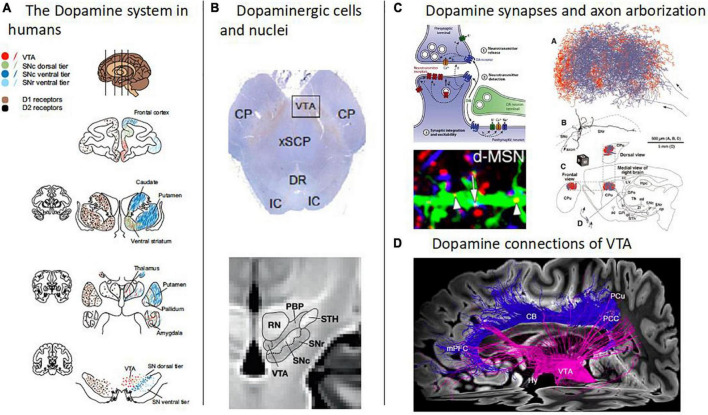
Overview of the human dopamine system. **(A)** The dopamine system in humans. Schematic depiction of the human brain’s dopamine system, captured by the distribution of both dopamine cell bodies in the midbrain, i.e., in substantia nigra pars compacta (SNc) and ventral tegmental area (VTA), and dopamine D1 and D2 receptors mainly in striatum and frontal cortices (adapted with permission from 12). Note the topographic arrangement of dopamine projections, indicated by the same colors at different levels of the dopamine system. **(B)** Dopaminergic cells and nuclei. Top: Dopaminergic cells in the midbrain (Tyrosine hydroxylase immunostaining) [adapted with permission from (168)]. Bottom: Boundaries between the red nucleus (RN), parabrachial pigmented nucleus (PBP), substantia nigra (SNc and SNr), and subthalamic nucleus, overlaid on a T1w template of midbrain MRIs with isotropic voxel size of 700 μm [adapted from (158), open-access]. **(C)** Dopamine synapses and axon arborization in mice. Top left: Scheme of the modulatory nature of dopamine synapses [adapted with permission from (13)]. Bottom left: Lattice-like, volume-focused distribution of dopamine synapses in the striatum of mice [adapted with permission from (14)]. Right: Dopamine axon arborization in the striatum of mice [adapted from (169), open-access]. **(D)** Dopamine connections of VTA. Diffusion-weighted MRI-based tractography of human VTA into the cortex [adapted with permission from (168)].

Schizophrenia is a disorder of behavior and brain that affects about 1% of the world population (1, 2). It is characterized by positive (e.g., delusions, hallucinations), negative (e.g., motivational deficits, anhedonia), and cognitive symptoms (e.g., impairments in cognitive flexibility or working memory). Schizophrenia shortens life expectancy by about 15 years and causes high socio-economic costs – for example, only around 20% of patients pursue a regular job (3–5). For decades, aberrant dopamine transmission has been proposed to play a central role in schizophrenia pathophysiology – this is called the “dopamine hypothesis of schizophrenia” (6, 7). Dopamine transmission, such as dopamine synthesis or dopamine receptor availability, is – at the molecular level – part of the brain’s distributed dopamine system. The dopamine system consists of broadcasting dopaminergic neurons, their axonal projections to the rest of the brain, and modulatory synapses of their axons in target regions (Figure 1). In the human brain, the cell bodies of dopaminergic neurons are usually organized in nuclei and are distributed almost exclusively in the brainstem, particularly in the midline and rostral portion of the mesencephalon (8, 9). Substantia nigra pars compacta (SNc) and ventral tegmental area (VTA) represent the two major dopaminergic nuclei in the brain. Based on *ex vivo* immunohistochemical quantification in humans, around 68% of all dopaminergic neurons identified in the mesencephalon were found in the SNc and 12% in the VTA (10). The axons of dopaminergic neurons project in a topographic manner more or less to the whole forebrain, with the highest density of dopaminergic synapses being found in the striatum (11, 12). Dopaminergic synapses are mainly modulatory and are laid out like lattices in target regions (13, 14).

The earliest versions of the dopamine hypothesis of schizophrenia relied on indirect evidence: for example, the clinical efficacy of antipsychotic drugs was found to correlate with their affinity to dopamine receptors (15). More recently, direct evidence has come from human *in vivo* molecular imaging studies of dopamine transmission, employing particularly positron emission tomography (PET) and single-photon emission computed tomography (SPECT) (7, 12). These methods have become the gold standard for investigation of the dopamine system. They are used to study distinct aspects of transmission at dopaminergic synapses (12), for example: (i) dopamine synthesis and storage in presynaptic axon terminals, using radioactively labeled precursors of dopamine that are able to cross the blood-brain-barrier, for example 18F-DOPA; (ii) postsynaptic dopamine receptor availability, using tracers that bind to D1 or D2/3 receptors; (iii) dopamine release from the presynaptic axon terminal, by measuring the displacement of these dopamine receptor tracers after administration of amphetamine (which induces dopamine release); and (iv) availability of transporters carrying dopamine from the synaptic cleft back into the presynaptic axon terminal, using tracers that bind to dopamine transporters.

Concerning the consistency of findings using these molecular imaging methods, several meta-analyses identified increased presynaptic dopamine synthesis and storage in the dorsal striatum as the most consistent aberrance of dopamine transmission in schizophrenia (16–18). Most studies included in these meta-analyses investigated patients with significant psychotic symptoms; during psychotic remission, in contrast, striatal dopamine synthesis and storage might be decreased (19–21). For other molecular imaging measures, such as availability of dopamine receptors and transporters, no consistent alterations have been identified (16, 22, 23); also, these outcome measures are more variable in patients than in healthy subjects, hinting at possible patient subgroups (24).

While molecular imaging methods have enabled great advances in the understanding of dopaminergic pathophysiology in schizophrenia and might even predict the course of symptoms (21), there are also several downsides of these methods.

First, molecular imaging can only investigate synaptic dopaminergic transmission. Other aspects of the dopamine system, such as the structure or connectivity of dopaminergic nuclei, are not analyzed. Furthermore, since the highest density of dopaminergic synapses is located in the striatum, signal quality of molecular dopamine imaging is usually highest in the striatum, whereas signals of extrastriatal regions are often unreliable (7, 12, 25). This also applies to substantia nigra: for example, concerning substantia nigra’s presynaptic dopamine synthesis and storage in schizophrenia, results have been inconclusive so far (26–28). Second, the interpretation of molecular imaging outcome measures is still debated: for example, the outcome of 18F-DOPA PET is interpreted as presynaptic dopamine synthesis and storage. 18F-DOPA is assumed to be metabolized to 18F-dopamine by the enzyme “aromatic acid decarboxylase.” However, normally, the enzyme “tyrosine hydroxylase,” which acts one step before aromatic acid decarboxylase, is the rate-limiting enzyme of dopamine synthesis (29). Therefore, 18F-DOPA PET outcome measures might not adequately assess presynaptic dopamine synthesis and storage. Third, PET has limited spatial and temporal resolution. The spatial resolution, usually in the range of a few millimeters, is limited in itself by the “traveling” of positrons over several millimeters before annihilation. The temporal resolution is limited by the use of graphical physiological modeling, which requires steady-state conditions that usually develop only after several minutes. In 18F-DOPA PET, for example, outcome measures are currently obtained only after 70 min of scan time at minimum. Fourth, scan costs are usually rather high, both due to tracer production and long scan times. Fifth, radiation exposure impedes repeated longitudinal examinations.

For these reasons, it is necessary to complement molecular imaging with other *in vivo* imaging tools to map the dopamine system and its changes in schizophrenia. Magnetic resonance imaging (MRI)-based methods that investigate distinct aspects of the dopamine system are promising candidates for such tools, at least for the study of further aspects of the dopamine system beyond synaptic transmission. In the following, we perform a scoping review on MRI of the dopamine system in schizophrenia; particularly, we will focus on four MRI methods which have been applied in schizophrenia: (i) neuromelanin MRI, which is thought to measure long-term dopamine function in SNc and VTA; (ii) morphometric MRI, which is assumed to measure the volume of SNc and VTA; (iii) diffusion MRI, which is assumed to measure fiber-based structural connectivity of SNc and VTA; and (iv) blood oxygen level-dependent (BOLD) functional MRI (fMRI) at rest, which is thought to measure blood oxygenation fluctuations of SNc and VTA and their functional connectivity, i.e., their correlations with other brain regions’ ongoing blood oxygenation fluctuations.

For each of these four methods, we initially describe its underlying signal, its outcome measures, and its downsides. Then, we present the current state of research in (i) schizophrenia, (ii) other psychiatric disorders – with a focus on disorders with similar (psychotic) symptoms, e.g., bipolar disorder or major depressive disorder, or with assumed dopaminergic abnormalities, e.g., substance use disorder –, and (iii) Parkinson’s disease, which is frequently used as a model for dopamine MRI due to dopamine cell loss in this disease.

After these method-specific paragraphs, we summarize and discuss the main findings as well as methodological issues and missing points within and across modalities. Furthermore, we outline questions for future research.

## Distinct magnetic resonance imaging-based approaches for studying the dopamine system in schizophrenia

### Neuromelanin magnetic resonance imaging

**FIGURE 2:**
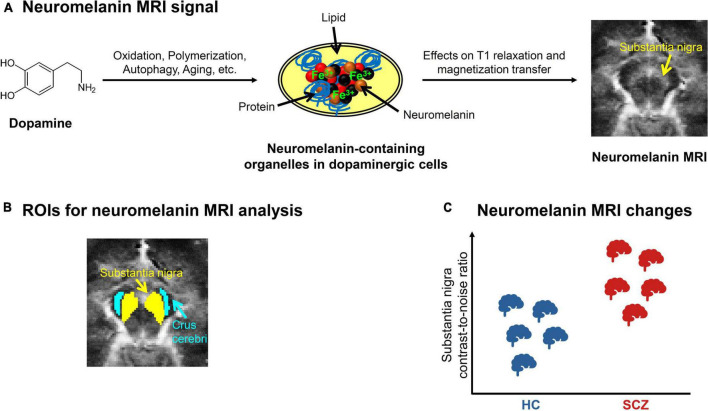
Neuromelanin MRI in schizophrenia. **(A)** Neuromelanin MRI signal. Schematic depiction of the metabolization of dopamine into neuromelanin-containing organelles and how neuromelanin influences the magnetic resonance imaging (MRI) signal. On the right, an exemplary preprocessed neuromelanin MRI image of one healthy subject is shown, with focus on the mesencephalon. **(B)** ROIs for neuromelanin MRI analysis. Typical regions of interest (ROIs) (43) used for contrast-to-noise-ratio analysis, which calculates the ratio between the MRI signals of substantia nigra and a reference region, e.g., crus cerebri. **(C)** Neuromelanin MRI changes in schizophrenia. Schematic depiction of the consistent elevation of substantia nigra contrast-to-noise-ratio in patients with schizophrenia (SCZ) compared to healthy controls (HC), based on meta-analytic evidence (40, 47).

Neuromelanin is a dark pigment produced by catecholamine metabolism. Over the life span, it gradually accumulates in the form of neuromelanin-iron complexes within organelles in the cell bodies of catecholamine-producing neurons, mostly in SNc, VTA, and locus coeruleus (Figure 2A) (30–33). It is thought to reflect a stable long-term marker of catecholamine metabolism, since it accumulates slowly and is not removed from neurons; its content in a given region only decreases after cell death, e.g., during neurodegeneration.

Since neuromelanin-iron complexes are paramagnetic (34), they shorten T1 relaxation and reduce magnetization transfer effects of nuclear magnetic resonance in tissues (Figure 2A) (35–38). These are probably the main factors contributing to neuromelanin-sensitive MRI. Neuromelanin MRI employs fast spin-echo or gradient-echo sequences, recently with direct magnetization transfer pulses, and shows neuromelanin-rich regions as hyperintense.

A typical outcome measure of neuromelanin MRI analysis is the contrast-to-noise ratio (CNR), which describes the ratio between the signal in a presumably neuromelanin-rich region, for example substantia nigra, and the signal of a reference region that presumably contains no neuromelanin, for example crus cerebri (Figure 2B) (39, 40). While neuromelanin MRI has been used to quantify dopaminergic neuron loss in neurodegenerative conditions for some time (39, 41, 42), it has recently also been validated to measure dopamine function *in vivo* in subjects without neurodegeneration: Cassidy et al. showed a relationship between substantia nigra CNR and both post-mortem neuromelanin concentration in the substantia nigra and amphetamine-induced striatal dopamine release measured by PET (43).

Downsides of the neuromelanin MRI signal comprise, first, the issue whether neuromelanin MRI specifically measures neuromelanin concentrations or also other substances/processes; further multimodal work combining imaging and histology is needed here. Second, although the spatial resolution of neuromelanin MRI (about 0.5 × 0.5 × 1.5 mm) is much higher than with PET, enabling the investigation of substantia nigra subregions (43), more refined investigations require improved sequences with better spatial resolution. For example, the ventral tegmental area is more difficult to image with neuromelanin MRI than the substantia nigra since it is smaller and contains less neuromelanin (43, 44). Furthermore, limited spatial resolution can lead to partial volume effects (45). Third, more work is needed to understand the temporal dynamics of neuromelanin accumulation, e.g., how fast one can expect signal changes when conditions (for example, psychotic symptoms) change. Fourth, the neuromelanin MRI signal could be confounded by many other non-structural factors, e.g., medication, about which reliable data are not yet available (40).

In patients with schizophrenia, studies have shown a reliable elevation of substantia nigra CNR (Figure 2C and Table 1), whereas for lcus coeruleus CNR, no clear trend has been observed. The first study by Shibata et al. reported a significantly increased substantia nigra CNR, but no significant alteration of locus coeruleus CNR (46). A more recent study by Cassidy et al. found increased substantia nigra CNR only in patients with strong psychotic symptoms (43). The current literature is summarized by two recent meta-analyses, which showed a consistent significant increase of substantia nigra CNR, but no consistent alteration of locus coeruleus CNR (40, 47). However, medication effects could not be assessed, since too few data were available. Furthermore, there are mixed results concerning the relationship between substantia nigra CNR and psychotic symptoms: one study observed a positive correlation (43), while others found no relationship (48, 49). Finally, longitudinal data or data from specific patient groups, e.g., psychotically remitted patients, are still lacking.

**TABLE 1:** Summary of dopamine MRI studies in schizophrenia.

Study	Patients/controls	Field strength	Technique	(Primary) outcome measure	Findings in patients
***Neuromelanin MRI***					
(46)	20/34	3T	FSE	CNR of SNc vs. SCP decussation	Increase
(48)	23/23	3T	FSE	CNR of SNc vs. SCP decussation	Increase
(173)	52/52	3T	3D-SPGR	CNR of SNc vs. midbrain tegmentum	Increase
(43)	33/30	3T	2D GRE-MT	CNR of SNc vs. CC	No difference (increase only in highly psychotic patients)
(51)	30/8	3T	FSE	Contrast ratio of SN	Increase
***Morphometric MRI***					
(69)	936/784	1.5T, 3T	3D MP-RAGE, SPGR	Whole-brain SBM, VTA	Increase
***Diffusion MRI and structural connectivity***					
(93)	24/22	3T	DWI; 4 b0s *b* = 1,300 s/mm^2^, 42 directions	Probability of belonging to fiber bundle and FA for VTA to NAcc, mOFC, lOFC, DLPFC and amygdala tracts	Increased probability of belonging to fiber bundle in patients for VTA-left amygdala and VTA-left mOFC tracts No difference in FA
(106)	22/23	3T	DWI; 8 b0s *b* = 900 s/mm^2^, 51 directions	Tract dispersion and FA of Striato-nigro-striatal tract	Increase tract dispersion in patients. No difference in FA.
(103)	46/44	3T	DWI; 4 b0s *b* = 1,300 s/mm^2^, 42 directions	FA of slMFB	Increase in severe psychotic affective patients compared to non-psychotic patients and controls
(92)	30/24	3T	DWI; 1 b0 *b* = 1,000 s/mm^2^, 12 directions	VTA/SNc Connectivity-based parcelation into limbic, prefrontal and sensorimotor parcels and evaluation to FA and streamline density index within these parcels	No difference in streamline density index nor FA in limbic, prefrontal sensorimotor parcels between groups
(104)	46/35	3T	DWI: 1b0 and *b* = 1,000 s/mm^2^, 32 directions	Connectivity index of VTA pathways to DLPFC, OFC, insular cortex	No difference
***Blood oxygenation MRI and functional connectivity***					
(145)	21/21 (UM-SCZ/HC)	3T	GRE-EPI, 3D MP-RAGE	VTA/midbrain seed-based FC	Decrease FC with precuneus, IPC, dACC, MCC, FG, IC, THAL, HPC, CAU, PU, cerebellum
(146)	26/22 (M-SCZ/HC)	3T	GRE-EPI, 3D MP-RAGE	VTA seed-based FC	Decrease FC with VLPFC, IC, LOC Increase with DLPFC
(147)	71/9/4 (M-SCZ/UM-SCZ/no records)	3T	GRE-EPI, 3D MP-RAGE	SN/VTA seed-based FC	Decrease with ACG, FG, CAU, OC, PAL, CAU, PU, PC/PCC, AG, TG, PG, PH, FG, OPER, CAL

ACG, anterior cingulate gyrus; AG, angular gyrus; CAL, calcarine cortex; CAU, caudate nucleus; CC, crus cerebri; CNR, contrast-to-noise ratio; D-SCZ, deficit schizophrenia; dACC, dorsal anterior cingulate cortex; DLPFC, dorsolateral prefrontal cortex; DWI, diffusion-weighted imaging; FC, functional connectivity; IPC, inferior parietal cortex; FG, fusiform gyrus; FG, superior, middle and inferior frontal gyri; FG, superior, middle and inferior frontal gyrus; GRE, gradient recalled echo; HC, healthy controls; EPI, echoplanar imaging; IC, insular cortex; LOC, lateral occipital complex; M-SCZ, medicated schizophrenia patients; MCC, middle cingulate cortex; mOFC, medial orbitofrontal cortex; lOFC, lateral orbitofrontal complex; MP-RAGE, magnetization-prepared rapid gradient-echo; MT, magnetization transfer; MTG, middle temporal gyrus; Nacc, nucleus accumbens; ND-SZ, non-deficit schizophrenia; OC, olfactory cortex; OPER, Rolandic operculum; PAL, pallidum; PC/PCC, precuneus/posterior cingulate cortex; PG, postcentral gyrus; PH, parahippocampal gyrus; PU, putamen; SC, structural connectivity; SCP, superior cerebellar peduncle; SN, substantia nigra; SNc, substantia nigra pars compacta; slMFB, supero-lateral medial forebrain bundle; TG, superior, middle and inferior temporal gyrus; THAL, thalamus; UM-SCZ, unmedicated schizophrenia patients (18 schizophrenia, 3 schizoaffective disorder); VLPFC, ventrolateral prefrontal cortex.

Regarding other psychiatric disorders, increased substantia nigra CNR has been shown for substance use disorder (50, 51). For major depressive disorder, there are only few studies so far: one study reported reduced locus coeruleus CNR, but no change in substantia nigra (46); another study found that neuromelanin MRI can be employed to visually discriminate depressed patients from healthy subjects (48); and a recent study reported that CNR of substantia nigra and ventral tegmentum correlates with gait speed in late-life depression (52). So far, we know of no studies conducting neuromelanin MRI in bipolar disorder.

In Parkinson’s disease, neuromelanin MRI has been widely used. The first study by Sasaki et al. observed reduced CNR of both substantia nigra and locus coeruleus in patients (39). Two recent meta-analyses showed that patients with Parkinson’s disease can be diagnosed with neuromelanin MRI-based measures (e.g., intensity or volume) with a sensitivity and specificity of more than 80%, respectively (41, 42).

### Magnetic resonance imaging-based morphometric analysis of dopaminergic nuclei

**FIGURE 3:**
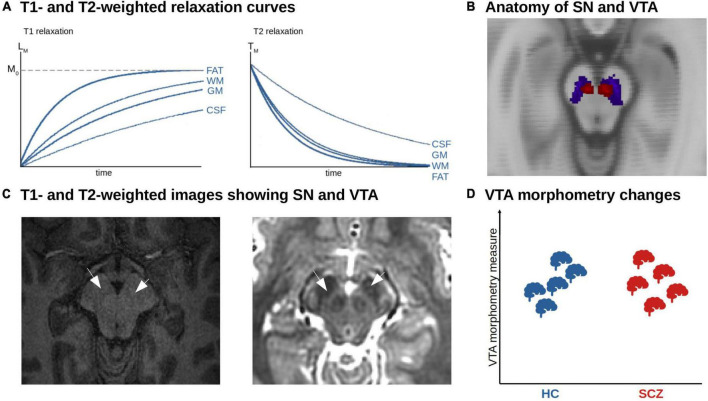
MRI-based morphometric analysis of dopaminergic nuclei in schizophrenia. **(A)** T1- and T2-weighted relaxation curves. The figure shows the recovery of the longitudinal magnetization (T1 relaxation). Tissues with a short T1 relaxation time such as fat and white matter (WM) recover faster and produce a stronger signal. The T2 relaxation reflects the decay of transverse magnetization. Tissue with a short T2 relaxation decays more quickly and produces a weaker signal. **(B)** Anatomy of SN and VTA. Axial view of the SN (blue) and VTA (red) derived from a probabilistic atlas shown on the MNI anatomical template (158). **(C)** T1- and T2-weighted images showing SN and VTA. Axial T1w (left) and T2w (right) structural imaging of the brainstem. The arrows point to the area of SN and VTA. **(D)** Unclear morphometric changes of dopaminergic nuclei in schizophrenia. Due to the low number of studies and methodological challenges, no clear trends have emerged so far.

For the analysis of the macrostructure of dopaminergic nuclei, structural magnetic resonance imaging based on T1- or T2-weighted sequences can be used (Figure 3A). These sequences are sensitive to differences regarding longitudinal (T1w) or transverse (T2w) relaxation time of free water magnetization across tissues (53). These signals can be used to estimate the volume of dopaminergic nuclei, which are anatomically defined using either manual delineation or automated techniques (Figure 3B).

Automated techniques can be divided into normalization-based and segmentation-based approaches (54). Concerning normalization-based approaches, whole-brain voxel-based morphometry (VBM) is a common tool. VBM uses non-linear spatial normalization (based on a template image) to transform T1-weighted images into standard space (55). Subsequently, each voxel’s probability of belonging to a standard gray matter mask is computed. The outcome of VBM is interpreted as voxel-wise gray matter density or volume (depending on the exact methodology). A downside of VBM is that regions such as substantia nigra or other brainstem nuclei, which are not part of standard gray matter masks due to their low contrast in T1w MRI, cannot be reliably investigated with this method; instead, the whole brainstem is segmented as white matter (56, 57).

Concerning segmentation-based tools, FreeSurfer is a common tool. FreeSurfer defines brain regions-of-interest on the subject level based on atlases and tissue borders (58, 59). The outcome of FreeSurfer is interpreted as the volume of a region-of-interest in mm^3^. A downside of FreeSurfer is that regions-of-interest have to be predefined in the software framework based on an atlas. For substantia nigra, for example, no such predefined region-of-interest exists, thus it cannot be investigated.

Taken together, a reliable automated segmentation of the substantia nigra and ventral tegmental area remains challenging (60, 61). This is mainly due to the low contrast of conventional T1-weighted MRI images in the brainstem. The rather high iron content in these brainstem nuclei shortens T1 and thus leads to a brighter contrast in the images, which makes it demanding to distinguish from white matter (Figure 3C) (62). Although probabilistic anatomical atlases for the substantia nigra and ventral tegmental area exist, region of interest-based analysis cannot be performed, as midbrain and brainstem are classified as white matter structure by common morphometry toolboxes, which also makes whole-brain analysis results in these areas questionable. Furthermore, post-mortem-based definitions of the human dopaminergic VTA/SN and their delineation in standard spaces such as MNI are not available at the moment – in contrast to the cholinergic basal forebrain, enabling VBM-based volumetric approaches (63, 64). FSL currently released a beta version of a toolbox called MIST, which is planned to provide tools to segment brainstem nuclei based on T1-weighted images in an upcoming release (65). Another possibility to segment nuclei in the brainstem and midbrain is an online processing pipeline called volBrain, which provides automated segmentation of deep gray matter nuclei, including substantia nigra, red nucleus, and subthalamic nucleus, based on T2-weighted images (66) – however, this is performed by the authors upon request, impeding large-scale applications.

For these reasons, manual segmentation remains the gold standard to delineate dopaminergic nuclei. Downsides of this approach are: it requires high expertise, is prone to inaccuracies, and is impracticable for applications to large cohorts (60, 61).

Further – more general – downsides of T1w-based morphometric MRI derive from its underlying signal. The MRI signal has a high sensitivity to common artifacts and non-structural factors, such as head motion, breathing effects, substance use, and body weight (67). In addition, observed morphometric changes, especially in the short term, might rely on perfusion changes instead of volume changes (68).

In schizophrenia, we are not aware of any region-of-interest-based studies that directly focused on VTA or SNc – therefore, no clear trend of alterations has emerged so far (Figure 3D and Table 1). However, a few whole-brain VBM studies reported morphometrically altered clusters that contained SNc and VTA. For example, Gupta et al. (69) investigated differences in gray matter concentration using source-based morphometry, which is a data-driven extension to VBM based on independent component analysis, and found increased gray matter density in the VTA in patients with schizophrenia. They found no association with the severity of positive or negative symptoms. Moreover, some meta-analyses of whole-brain VBM studies in schizophrenia reported altered VBM scores in brainstem clusters partly covering VTA/SNc (70, 71). For example, across 127 VBM studies including over 13.000 patients and healthy controls, we observed reduced gray matter volume in a cluster located mainly in the brainstem but partly overlapping with SNc (71). The interpretation of such findings, however, is obviously difficult since these meta-analyses were not focused on dopaminergic nuclei as regions of interest.

Concerning other psychiatric disorders, e.g., bipolar disorder, major depressive disorder, or substance use disorder, we are not aware of any direct morphometric studies of dopaminergic nuclei either. Although some voxel-based whole-brain analyses revealed a morphometric increase in substantia nigra or midbrain in bipolar and major depressive disorder, and a decrease in substance use disorder [e.g., (72–74)], coordinate-based meta-analyses did not show consistent effects in SNc or VTA across studies (75, 76).

The few studies in psychiatric diseases are in stark contrast to the research in Parkinson’s disease, where several studies about morphometric differences of the dopaminergic nuclei have been performed so far. For example, Geng et al. (77) manually segmented the substantia nigra based on proton density- and T2-weighted images and did not find any alterations in different stages of Parkinson’s disease. A meta-analysis on substantia nigra volumetry reported a significant decrease (78). However, the included studies were heterogeneous regarding image processing and anatomical labeling of the substantia nigra.

### Diffusion magnetic resonance imaging-based analysis of structural connectivity of dopaminergic nuclei

To date, diffusion-weighted imaging (DWI) is the only technique available to study white matter fibers and tracts *in vivo* in humans. DWI relies on the principle that water molecules are in constant motion. In free water (i.e., water molecules not bound to, for example, macromolecules), molecules can equally move or diffuse in all directions – this is called isotropic diffusion. In biological tissues comprising certain cell architectures, the diffusion of water molecules is hindered by cell membranes, thus resulting in anisotropic diffusion (79–81). Thus, by measuring the orientational dependence of water diffusion, it is possible to estimate the orientation of underlying membranes, for example axons, and to delineate fiber pathways (82, 83). DWI is sensitive to water diffusion by applying magnetic field gradient pulses in different directions (b-vectors; minimum 6, usually 32 to 64 in clinical settings), in order to map water diffusion not only for a spectrum of directions but also for each voxel across the whole brain (79–81). The gradients’ strength and timing is defined by the so-called *b*-value: the higher the *b*-value, the stronger the diffusion effect.

**FIGURE 4:**
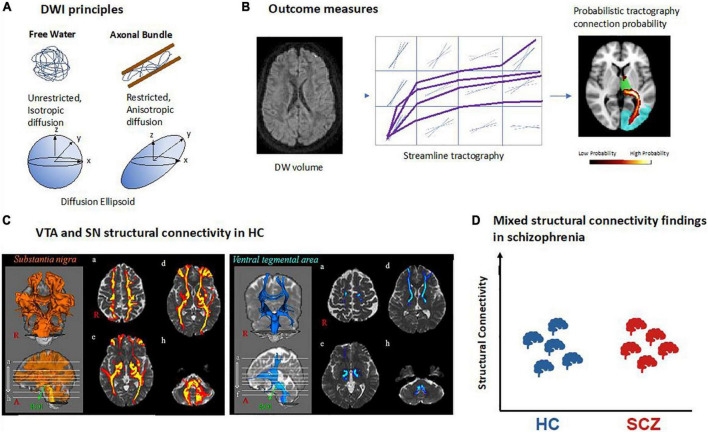
Structural connectivity of dopaminergic nuclei in schizophrenia based on diffusion-weighted MRI. **(A)** Principles of diffusion-weighted imaging (DWI). Water molecules are free to diffuse in all directions in free water while their diffusion is restricted in biological tissue such as axonal fiber bundles and will follow their main orientation. It can be quantified using a mathematical model called a tensor which can be represented as an ellipsoid with its main axis representing the principal direction of diffusion. In the case of free water there is no principal direction of diffusion thus the ellipsoid takes the shape of a sphere. **(B)** Structural connectivity outcome measures. Example of a DWI volume. All DWI volumes are used to perform tractography between two regions of interest. In deterministic tractography, each reconstructed streamline follows the main direction of diffusion per voxel or while in probabilistic tractography as represented here, a distribution of possible orientations is modeled and each streamline is reconstructed from each possible orientation. In the reconstructed path, the value in each voxel represents how many streamlines passed through it. The higher number of streamlines per voxel, the higher the probability for that voxel to be part of a fiber bundle. **(C)** VTA and SN structural connectivity in healthy controls (HC). Example of probabilistic tractography from VTA and SN showing their connectivity to the rest of the brain [adapted with permission from (107)]. **(D)** Mixed structural connectivity findings in schizophrenia. Structural connectivity can be estimated by a variety of different outcome measures, making comparisons across studies difficult; so far, no clear trends have emerged.

Magnitude and directionality of water diffusion can be modeled by so-called 3D diffusion tensors (DTI, diffusion tensor imaging). In mathematical terms, a voxel-wise diffusion tensor is a 3 × 3 matrix, which subjects to diagonalization and results in a set of three eigenvectors. Such a tensor can be visualized as an ellipsoid, for which the three eigenvectors represent the major, medium, and minor axis of the ellipsoid, respectively, and the corresponding three eigenvalues represent the apparent diffusivities along these axes (Figure 4A). From the tensor, one can estimate both the direction of maximum diffusivity and the diffusivity in any arbitrary direction (84). Several measures are derived from the diffusion tensor, such as fractional anisotropy (FA), which describes how strongly the diffusion is pronounced to one main direction (85).

While FA can inform about the microstructure of white matter, it does not provide information about pathways of white matter, for example tracts connecting two brain regions such as VTA and parts of the prefrontal cortex. However, 3D representations of fiber bundles can be obtained by identifying streamlines of adjacent primary eigenvectors of diffusion across neighboring voxels (86). This procedure is called tractography, and its output is called structural connectivity. In other words, streamlines can be defined as the adjacent sets of 3D points produced by tractography algorithms (87). There are two main types of tractography: In deterministic tractography (88), each streamline is generated following the main direction of diffusion of each voxel step by step, until a too wide curvature angle or a low signal stops the pathway. Probabilistic tractography (89), in contrast, considers the uncertainty of the main orientation of diffusion and estimates a distribution of potential orientations; next, a streamline is generated following each of these possible orientations. The higher the number of streamlines per voxel, the higher the probability for that voxel to be part of a fiber bundle (Figure 4B). Using probabilistic tractography, it is possible to parcellate seed regions according to predefined target regions, which are known to project into the seed, by the highest number of streamlines into the seed region (90, 91).

Several limitations of DWI and DTI-based tractography make the study of dopaminergic connectivity challenging. First, DWI is typically of low spatial resolution (i.e., about 2 × 2 × 2 mm), which makes tractography of small regions such as VTA difficult. Therefore, some studies have opted for manually delineating these structures (92) or drawing small spheres around brain coordinates of VTA/substantia nigra with subsequent tractography from there (93, 94). Second, tractography is unspecific to the type of neuronal projections, i.e., one cannot differentiate, for example, between dopaminergic and glutamatergic connections from VTA. Third, microstructural measures of tracts such as FA are unspecific to microscopic features, reflecting a wide range of processes or structures such as myelin content, axonal packing, axonal diameter, or gliosis (95). Thus, there is a need for more specific methods, for example, myelin water imaging (96) or neurite orientation dispersion and density imaging (97). Fourth, tractography is not sensitive for the direction of tracts, efferent or afferent relative to a seed region (95, 98). Fifth, tractography is unreliable in regions where fibers have more than one main orientation, such as regions of crossing or fanning fibers (99–101). To overcome this limitation, more accurate models were developed, for example constrained spherical deconvolution (CSD), which is used to estimate the distribution of fiber orientations within each imaging voxel, regardless of the number of underlying fiber orientations (102). Finally, there is a wide range of distinct measures obtained from both DWI and DTI-based tractography, which are – confusingly – “lumped together” under the label “structural connectivity,” making comparisons across studies challenging. Examples of outcome measures comprise the number of streamlines per voxel; the percentage of streamlines starting from the seed region (for example VTA) that reach the target region (for example orbitofrontal cortex), also referred to as connectivity index; the volume of streamlines in the seed region divided by the total volume of the seed region [also called the streamline density index (92)]; or the probability for a given voxel to be part of an *a priori* defined tract connecting two regions (93). Changes in any of these measures for the pathway connecting VTA and orbitofrontal cortex, for example, would indicate alterations in this pathway; however, the exact underlying biological process (number of axons, degree of compaction of axons, etc.) remains unclear. In addition, measures of white matter microstructure can also be referred to as structural connectivity, as is the case for FA (103, 104) or for tract dispersion index (105), an index of fiber geometry which reflects the degree to which tracts deviate from being parallel such as in fanning fibers, for example (106). As for previously described measures of structural connectivity, FA changes in the VTA-orbitofrontal cortex connection might reflect several processes such as axonal degeneration, demyelination, gliosis, or characteristics of fiber architecture such as kissing or fanning fibers as would also be indicated by the tract dispersion index.

Several studies have investigated both substantia nigra and VTA structural connectivity in healthy humans. Most studies performed either DTI-based or CSD-based probabilistic tractography with seeds in the VTA or SN, respectively (91, 107, 108). An alternative approach is, first, performing whole-brain tractography and then selecting the superolateral part of the medial forebrain bundle (MFB) which connects the VTA to both frontal lobe and forebrain (109–111) to specifically target connections between VTA and parts of the prefrontal cortex such as the orbitofrontal cortex (112). Finally, one study compared substantia nigra and VTA connectivity among each other, observing higher scores of substantia nigra connectivity for several brain regions including primary motor and somatosensory cortex, premotor cortex, and caudate nucleus (Figure 4C) (107). The most detailed study of substantia nigra connectivity to date is probably the one by Zhang and colleagues, who used probabilistic tractography and confirmed in humans a tripartite connection organization that was described before in monkeys by the use of similar techniques: the medial substantia nigra connects with limbic striatal and cortical regions, the ventral substantia nigra with associative regions of cortex and striatum, and the lateral substantia nigra with somatomotor regions of striatum and cortex (91).

Few studies have investigated VTA/substantia nigra structural connectivity in schizophrenia so far, each using a different measure of structural connectivity. Thus, findings are rather mixed, and no clear pattern has emerged so far (Figure 4D and Table 1). Bracht and colleagues (93) performed DTI-based probabilistic tractography from VTA to ventral striatum and prefrontal cortex. They found higher probability values for belonging to certain fiber bundles in schizophrenia patients compared to healthy controls in both the left VTA-to-orbitofrontal cortex and left VTA-to-left amygdala pathway. These probability values of both pathways were associated with patients’ negative symptom severity (measured by the negative sub-scale of positive and negative syndrome scale, PANSS). Basile and colleagues used CSD-based tractography to perform a connectivity-based parcelation of SN/VTA regions based on their connections to limbic, prefrontal, and sensorimotor cortices (92). Between patients and controls, they observed no difference in the parcelation into “limbic,” “prefrontal,” and “sensorimotor” territories across groups, and additionally no differences in both streamline density index and FA in these territories. Using connectivity index and FA as measures of structural connectivity between VTA and dorsolateral prefrontal cortex, orbitofrontal cortex, and insular cortices, Giordano and colleagues reported no differences between patients and controls either (104). However, two other studies, which used a whole-brain tractography approach first and then identified pathways of interests such as the striato-nigro-striatal pathway or the medial forebrain bundle including VTA/substantia nigra connections, reported mixed findings (103, 106): altered tract dispersion index was found in patients with schizophrenia (106), while the medial forebrain bundle FA was unchanged in most patient subgroups of schizophrenia spectrum except for those with severe psychotic symptoms – they had increased FA values compare to both patients with less psychotic symptoms and healthy controls (103).

In other psychiatric disorders, most studies investigated the microstructure of the medial forebrain bundle. For example, in major depressive disorder, Bracht and colleagues used a bilateral probabilistic fiber tracking approach to extract pathways between the VTA and ventral striatum and medial and lateral prefrontal cortex, respectively, and compared mean FA values (93) between patient subgroups and healthy controls. They found reduced FA only in patients during a severe depressive episode. In bipolar disorder, using a similar methodology, Denier and colleagues did not find any FA differences (113). Regarding substance use disorders, lower FA in the VTA-Nucleus accumbens tract as part of the medial forebrain bundle has been reported in individuals with stimulant use disorders (114), and lower FA in several white matter tracts has been reported in adolescents and young adults with a history of cannabis use (115) as well as in cocaine, nicotine and alcohol users (116).

In Parkinson’s disease, lower FA (117) and reduced structural connectivity concerning several measures such as streamline density were reported for nigrostriatal projections in patients with Parkinson’s disease (118, 119).

### Blood oxygenation magnetic resonance imaging of dopaminergic nuclei and their blood oxygen level-dependent resting-state functional connectivity

**FIGURE 5:**
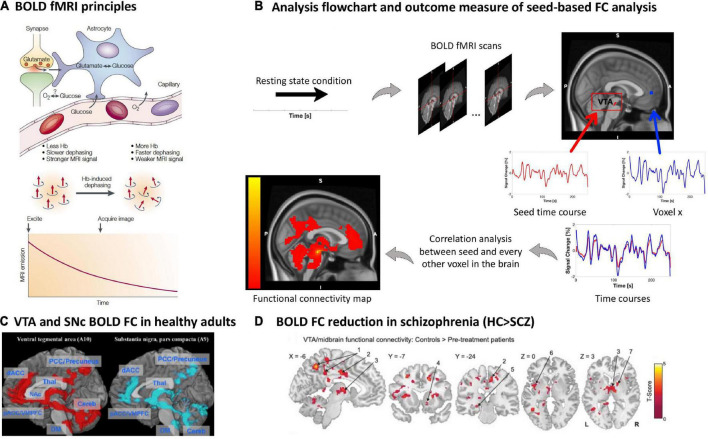
Blood oxygenation MRI of dopaminergic nuclei and their BOLD resting-state functional connectivity in schizophrenia. **(A)** BOLD fMRI principles. The figure shows the proposed relationship between neurophysiological underpinnings of BOLD signal fluctuation along time, i.e., synaptic activity, neurotransmitter recycling and metabolic demand (above), and the physical underpinning of the T2*-weighted imaging-based effect of deoxyhemoglobin on the MRI signal (below) [adapted with permission from (121)]. **(B)** Analysis flow chart and outcome measure of seed-based FC analysis. To investigate the functional architecture of the dopaminergic system during resting state, a region of interest is selected as a seed, for example the VTA. The delineation of the seed can be done *via* manual segmentation, created based on a specific MNI coordinate or based on a mask derived from an atlas, e.g., as in this example (170). Next, a correlation coefficient (e.g., Pearson’s) is calculated between the seed’s time course and every other voxel in the rest of the brain, creating individual seed-wise FC maps as outcome (125, 126). **(C)** VTA and SNc BOLD FC in healthy adults. Positive whole-brain resting state FC analysis of the VTA and SNc. All comparisons were thresholded at an FWE-corrected voxel-level significance of *p* < 0.05 and at an FDR-corrected cluster-level significance of *p* < 0.05. pACC, perigenual anterior cingulate cortex; dACC, dorsal anterior cingulate cortex; PCC, posterior cingulate cortex; VMPFC, ventromedial prefrontal cortex; NAc, nucleus accumbens; THAL, thalamus; Cereb, cerebellum; DM, dorsal medulla [adapted with permission from (171)]. **(D)** BOLD FC reduction in schizophrenia. Regions showing VTA/midbrain connectivity in controls (*n* = 21) compared to pre-treatment unmedicated patients with schizophrenia (*n* = 21); two tailed two sample *t*-test corrected with a false discovery rate (FDR) at *p* < 0.05 [adapted with permission from (145)].

Blood oxygenation reflects the amount of oxygenated hemoglobin of erythrocytes in the blood. Blood oxygenation level dependent functional magnetic resonance imaging (BOLD fMRI) is a neuroimaging technique that non-invasively depicts local changes of blood oxygenation along time (120). First, concerning physical underpinnings of the BOLD signal, de-oxygenated hemoglobin has paramagnetic properties, leading to altered decay of transversal magnetization of free and hemoglobin-bound water (i.e., H + -nuclei of water molecules) under MRI conditions (T2*-weighted imaging, gradient-echo echo-planar imaging) (121, 122). Fast sampling rates enable mapping of changes in T2* relaxation of water magnetization in a certain voxel, which are then interpreted as BOLD changes along time, with temporal and spatial resolutions between 0.5–3 s and 2–3 mm, respectively (Figure 5A). Second, concerning neurophysiological and physiological underpinnings of BOLD changes, blood oxygenation is a summary measure reflecting a couple of processes, namely changes in neuronal activity and neurovascular coupling processes, which link neuronal activity with blood oxygenation. Neurovascular coupling includes hemodynamic processes (e.g., blood volume and flow changes), vascular processes (e.g., vascular reactivity based on myocytic and endothelial activity), and mediating processes (e.g., astrocytic activity) (123) (Figure 5A). In the human cortex, the BOLD signal comes mainly from perforant and branching arterioles and venules (123). In gray matter nuclei such as VTA/SNc, BOLD changes reflect changes in the neuronal and neurovascular processes mentioned above, including those directly related to dopaminergic cells and their local cellular support network. Already from this short description, one can infer that the mapping of VTA/SNc BOLD fluctuations onto dopaminergic neuronal activity is rather unspecific, weak, and limited by fMRI’s temporal and spatial resolution.

During resting states, the spontaneous BOLD fMRI signal fluctuates in an infra-slow frequency range, 0.01 to 0.15 Hz (124). The correlation between spontaneous BOLD fluctuations of different regions or voxels, e.g., between seed regions such as VTA/SNc and cortical regions of the forebrain, can be studied *via* pair-wise correlation coefficients (mainly normalized Pearson’s correlation coefficients) (125, 126) (Figure 5B). Correlation coefficients are interpreted as BOLD functional connectivity (FC) of ongoing brain activity and are typically used to investigate seed-based FC patterns, reflecting the seed’s static functional connectivity architecture (124, 127) (Figures 1D, 5C). Particularly, FC patterns of VTA/SNc have been used to investigate the functional architecture of the dopaminergic system with the assumption that any BOLD fluctuation coherent (or correlated) to the VTA/SNc time course might be related to dopaminergic projections from the VTA/SNc toward, for example, the forebrain (128, 129). The resulting FC pattern in healthy participants indeed overlaps largely with patterns of tracer-based anatomical dopaminergic projections identified in primates, including projections to the ventral striatum, amygdala-hippocampal complex (130), prefrontal and cingulate cortices (130, 131), as well as thalamus (132) (Figures 1D, 5C). Furthermore, one should note that BOLD FC is indeed sensitive to external dopaminergic challenges, suggesting that changes in BOLD FC might reflect changes in dopamine transmission (133–136).

However, potential downsides of the BOLD fMRI signal, BOLD FC in general, and BOLD FC of the VTA/SNc in particular have to be mentioned. In general, BOLD fMRI is sensitive to several confounds, including, for example, scanner-related artifacts such as hardware-related instabilities and undesired variations in the magnetization field, scanning-dependent low frequency drifts, and subject-induced motion artifacts, making multi-scanner or multi-site studies challenging (137). Specifically related to VTA/SN, cardiac and respiratory-related artifacts, such as arterial pulsation, are important in the brainstem due to its proximity to large pulsing vessels (138–141). Additional challenges concern the preprocessing of images, specifically the co-registration process, where BOLD-related T2*-weighted images with a limited spatial resolution (i.e., voxel size ~2 × 2 × 2 mm) are mapped to high-resolution T1-weighted structural images for anatomical reference (i.e., ~1 mm) causing possible spatial distortions, mismatched alignment, and partial volume effects in brainstem structures. Although there are several advanced scanning and preprocessing procedures accounting for these limitations (e.g., high-resolution fMRI with stronger magnetic fields such as 7T MRI, movement-induced artifact detection and application of correction algorithms) (142–144), BOLD FC patterns for VTA/SNc seeds should be evaluated carefully.

In patients with schizophrenia, studies of VTA/substantia nigra resting-state BOLD FC demonstrated an overall trend to reduced functional connectivity between VTA/substantia nigra and forebrain structures in patients (145–147). Hadley and colleagues were the first to study VTA BOLD FC in patients with schizophrenia or schizoaffective disorder. Patients were initially unmedicated and received antipsychotic treatment with risperidone during the study. They were scanned before start of the treatment, after 1 week, and after 6 weeks, while symptom severity was assessed weekly. The seed for the analysis was defined as a 3-mm spherical region of interest at Montreal Neurological Institute (MNI) coordinates [0; 16; −7] representing the VTA/midbrain (148). Before the start of treatment, unmedicated patients showed reduced VTA/midbrain FC toward several cortical and subcortical regions compared to controls (145) (Figure 5D and Table 1). After 1-week treatment, connectivity toward the thalamus was restored; however, connectivity strength was not correlated to treatment response. Interestingly, pre-treatment connectivity strength toward the dorsal anterior cingulate cortex was positively correlated with a good response to treatment, whereas pre-treatment strength toward areas of the so-called “default mode network” demonstrated a negative correlation with treatment response (145). With a similar VTA definition, this pattern of widespread reduced VTA FC was largely replicated by Giordano and colleagues in medicated clinically stable patients (146), and later by Xu and colleagues using a VTA/substantia nigra seed in both medicated and unmedicated patients (147). This FC reduction was correlated with patients’ motivational deficits (146, 147). In contrast, increased resting-state BOLD FC in schizophrenia was observed only by Giordano and colleagues, namely hyperconnectivity between the VTA and the dorsolateral prefrontal cortex, interpreted as an imbalance between the so-called “salience” and “executive central” resting-state networks (146). A fourth study did not find any significant differences regarding VTA connectivity between patients and controls; around 60% of the sample were chronic patients (149).

In other psychiatric disorders, VTA/SNc resting-state BOLD FC results are mixed. For instance, regarding substance-related and addictive disorders, reduced connectivity between substantia nigra and caudate nucleus as well as between VTA and medial orbitofrontal cortex, anterior cingulate cortex, and nucleus accumbens has been observed in opioid use disorder (150), while reduced connectivity of VTA with olfactory tubercle, medial orbitofrontal cortex, and nucleus accumbens was found in patients with internet gaming disorder (42, 151). In both major depressive disorder and bipolar disorder, concerning VTA FC, studies observed reduced connectivity with frontal regions (149, 152), cerebellum and mediodorsal thalamus (152) in patients, increased connectivity with pre- and postcentral gyrus, frontal and temporal gyrus, occipital lobe, pons (152), limbic regions (149, 152), cerebellum, and posterior cingulate cortex (149), or no changes between groups of patients and controls (153). Concerning substantia nigra FC in major depressive disorder patients, both reduced connectivity with anterior cingulate cortex and cerebellum and increased connectivity with pre- and postcentral gyrus, insula, inferior frontal gyrus, superior temporal gyrus, parahippocampus, and occipital lobe have been reported (152).

In Parkinson’s disease, the only study so far observed reduced FC of substantia nigra with thalamus, pallidum, caudate, putamen (154).

## Discussion: Current research gaps and potential developments in the field

We performed a scoping review of four MRI methods imaging the dopamine system in schizophrenia, namely neuromelanin MRI, morphometric MRI, diffusion MRI, and BOLD resting-state fMRI. We set these studies in context regarding methodological underpinnings (with focus on the imaging signal and its downsides), typical results in healthy controls, and aberrances in both other psychiatric disorders frequently comprising psychotic symptoms and Parkinson’s disease, the reference disorder for MRI-based dopamine system imaging. A sufficient number of studies with homogeneous methodology (five) was identified only for neuromelanin MRI (Table 1), for which meta-analyses have already been conducted. For the other three modalities, the number of studies was limited (e.g., morphometric MRI) and/or studies were quite heterogeneous in terms of outcome measures or patient groups (e.g., diffusion MRI) (Table 1). In the following, we first give an overview of the included methods and what aspects of the dopamine system they measure; second, we discuss findings of dopamine MRI in schizophrenia for each imaging modality regarding homogeneity, signal downsides, findings in other disorders, and relation to symptoms; third, we outline missing points and limitations across modalities; finally, we outline some questions for future research.

### Summary of modalities and their coverage of the dopamine system

While molecular imaging, e.g., PET, of the dopamine system in schizophrenia focuses on aberrant dopamine transmission at the symaptic level, MRI-based imaging methods complementarily focus on other parts of the dopamine system (Figure 1): neuromelanin MRI focuses on cumulative catecholamine metabolism in dopaminergic nuclei, morphometric MRI on the structure of dopaminergic nuclei, diffusion MRI on structural tract-based connections of dopaminergic nuclei, and BOLD resting-state fMRI on functional blood oxygenation-based connectivity of dopaminergic nuclei. Thus, the four methods investigate different but complementary aspects of the dopaminergic system. Neuromelanin and morphometric MRI measure localized processes within dopaminergic nuclei, while BOLD fMRI measures signal coherence between dopaminergic nuclei and other brain regions. Diffusion MRI, on the other hand, can measure microstructure within dopaminergic nuclei, but also the macro- and microscopic architecture of fibers/tracts in regions rather distant from dopaminergic nuclei, which presumably contain fibers from/to dopaminergic nuclei.

### Findings in schizophrenia for each modality, comparison to other disorders, and problems

Neuromelanin MRI studies showed a consistent increase of contrast-to-noise-ratio in the substantia nigra in patients with schizophrenia (40, 47) (Figure 2). This is interpreted as increased accumulation of neuromelanin in the substantia nigra, which is an indicator of increased long-term catecholamine metabolism. The methodology across studies was rather homogeneous – only the reference regions for contrast-to-noise ratios varied. Downsides of the signal comprise limited knowledge about the specificity of the signal for neuromelanin, influences from factors such as medication, limited spatial resolution, and the very low temporal resolution (presumably in the range of years). Concerning substantia nigra contrast-to-noise ratio in other disorders, there is a similar trend for increase in substance use disorder, no clear trend in major depressive disorder, no studies in bipolar disorder, and a clear trend for reduction in Parkinson’s disease. There is no clear trend for a link with psychotic symptoms; longitudinal studies are lacking.

The few morphometric MRI studies of dopaminergic nuclei showed no clear trend for morphometric alterations in SNc and VTA in patients with schizophrenia (Figure 3): while the study of Gupta and colleagues (69) demonstrated increased volume in the brainstem overlapping with SNc, using whole-brain VBM in a large patient sample (>900), the meta-analysis of whole-brain VBM studies in schizophrenia by Brandl et al. (71) found reduced VBM scores in a similar brainstem cluster. As a major limitation of these approaches, it has to be noted that we identified no study directly focusing on dopaminergic nuclei (e.g., with a ROI-based approach); instead, we only identified whole-brain studies/meta-analyses of T1-based morphometry that observed changes overlapping with dopaminergic nuclei. Downsides of the signal include the unclear biological underpinnings (particularly regarding the temporal dynamics of the signal), no specificity for dopaminergic neurons, low contrast in dopaminergic nuclei, which impedes automatic delineation and necessitates manual segmentation with ensuing problems, as well as sensitivity to artifacts. Concerning morphometric MRI measures of dopaminergic nuclei in other disorders, there is a trend for increase in major depressive disorder and bipolar disorder, a trend for decrease in substance use disorder, and consistent decrease in Parkinson’s disease. In schizophrenia, the relationship with symptoms is unclear. We know of no longitudinal studies in schizophrenia.

Diffusion MRI studies in schizophrenia showed alterations in several structural connectivity measures, hinting at an aberrant macroscopic fiber architecture and fiber microstructure. However, due to the considerable heterogeneity of methods and measures, no clear pattern has emerged so far (92, 93, 103, 104, 106) (Figure 4). Further downsides of the signal include low spatial resolution, no specificity regarding dopaminergic neurons, unclear underlying microscopic processes, inability to determine the directionality of fiber tracts, and limited reliability in areas with diffuse fiber orientations. Concerning structural connectivity of dopaminergic nuclei in other disorders, there is a trend for reduction in major depressive disorder and substance use disorder, no clear trend in bipolar disorder, and a trend for reduction in Parkinson’s disease. In schizophrenia, despite inconsistent findings, at least one study suggested stronger aberrances in patients with more psychotic symptoms. We are not aware of any longitudinal studies.

Resting-state BOLD fMRI studies in schizophrenia showed a trend for reduced resting-state functional connectivity of dopaminergic nuclei with widespread forebrain areas (145–147) (Figure 5). However, results were heterogeneous – some studies reported no group differences or even increased functional connectivity –, making conclusions difficult. Downsides of the signal include the lack of specificity for dopaminergic neurons, influences from non-neuronal sources, limited spatial resolution, sensitivity to artifacts (e.g., motion), and varying processing methods. In schizophrenia, the link to symptoms or longitudinal changes have not been investigated so far, to our knowledge. Concerning functional connectivity of dopaminergic nuclei with various subcortical and cortical regions in other disorders, both decreases and increases were observed in major depressive disorder and bipolar disorder, and a trend for reduction in substance use disorder and Parkinson’s disease. In schizophrenia, there are hints at links between dopaminergic nuclei functional connectivity at rest and both positive and negative symptoms of schizophrenia. One longitudinal study showed a correlation between resting-state functional connectivity alterations and treatment response.

### Overarching problems across modalities

**FIGURE 6:**
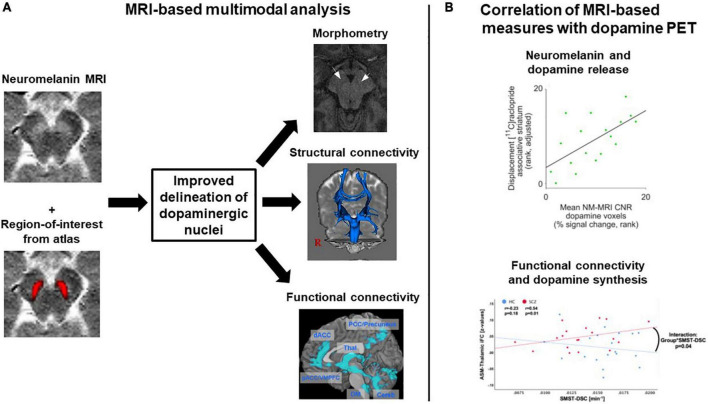
Exemplary concepts for multimodal analyses involving several dopamine MRI methods or combining MRI and dopamine PET. **(A)** MRI-based multimodal analysis. A combination of neuromelanin MRI data, which have a high contrast for dopaminergic midbrain nuclei, and regions-of-interest from atlases, which for example were constructed based on structural MRI data, might improve the delineation of dopaminergic nuclei. The regions-of-interest derived from such a delineation procedure could then, in a second step, be used for morphometric analyses or as seed regions for structural and functional connectivity analyses. Plots adapted with permission from (171) and (107). **(B)** Multimodal combination of MRI and dopamine PET data. Two examples from the literature are shown, displaying correlation analyses between MRI measures and dopamine PET measures. Plots adapted under Creative Commons license or re-use of own work in line with journal guidelines from (43) and (172). ASM, auditory-sensorimotor network; HC, healthy controls; iFC, intrinsic functional connectivity; NM-MRI CNR, neuromelanin MRI contrast-to-noise-ratio; SCZ, patients with schizophrenia; SMST-DSC, sensorimotor striatum dopamine synthesis capacity.

First, neither morphometric MRI nor diffusion MRI nor BOLD fMRI are specific for dopaminergic neurons. In contrast, neuromelanin MRI is assumed to be rather specific for catecholaminergic neurons, and with sufficiently accurate delineation of dopaminergic nuclei, it can be assumed to have good specificity for dopaminergic neurons. To test and potentially increase the specificity of dopamine MRI modalities, multimodal studies are necessary, combining dopamine MRI modalities with either dopamine PET, the gold standard for *in vivo* dopamine imaging, – for example in the form of simultaneous PET/MRI – or neuromelanin MRI, as the most specific dopamine MRI approach (Figure 6). To our knowledge, only one multimodal study combined neuromelanin MRI with dopamine release PET (and additionally with arterial spin labeling-based perfusion MRI) in a combined sample of healthy subjects and patients with schizophrenia (43). They reported a link between neuromelanin contrast-to-noise-ratio and dopamine release in the associative striatum; however, no effect of schizophrenia on this link was found. In Parkinson’s disease, on the other hand, a recent study reported a patient-control difference concerning the link between neuromelanin MRI and dopamine synthesis and storage, measured by 18F-DOPA PET (155). Further systematic multimodal studies in patients with schizophrenia are needed, also encompassing other modalities. Naturally, such approaches are only correlative, i.e., they cannot determine whether observed correlations are direct or mediated *via* further processes. If no correlations were to be observed, on the other hand, this would not preclude links with other aspects of the dopaminergic system currently not accessible to dopamine MRI.

Second, the number of studies in general and the level of systematic investigations in particular was low. For example, we identified only one study of morphometric MRI in dopaminergic nuclei, whereas there is a large number of studies in Parkinson’s disease, which shows the general feasibility of this approach. Regarding diffusion MRI, on the other hand, there is a plethora of different outcome measures, which makes comparisons very difficult. Furthermore, more studies with a precise clinical characterization of patient samples, e.g., regarding symptom status or medication, are needed. Most studies included in this review reported only limited patient characteristics. For example, the current disease state is usually not sufficiently reported – e.g., are patients currently in a psychotic episode or in remission of psychotic symptoms? Future studies need to characterize investigated patients more thoroughly. Longitudinal approaches could clarify how measures change over time and with changing symptom status, as has been done for other MRI measures [e.g., for thalamocortical structural connectivity see (156)]. For neuromelanin MRI, for example, Cassidy et al. found no difference in substantia nigra contrast-to-noise-ratio between patients at high risk for psychosis and patients with diagnosed schizophrenia (43). Such approaches should be extended to longitudinal measurements within the same patients with schizophrenia, e.g., from psychotic phases to psychotic remission. Moreover, it is incompletely understood how dopamine MRI measures are influenced by antipsychotic drugs, whose clinical effects greatly rely on their blockade of dopamine receptors. More studies comparing medicated and unmedicated patients are necessary. Finally, also studies contrasting treatment-responsive with treatment-resistant patients should be conducted, to further elucidate mechanisms of resistance to antipsychotic drugs. This has already been done in multimodal MRI-PET studies, for example combining 18F-DOPA PET-based striatal dopamine synthesis capacity with prefrontal gray matter volume (157) or frontostriatal functional connectivity (21). Future studies should also apply these approaches to MRI methods focusing on dopaminergic midbrain nuclei.

Third, it remains challenging to accurately delineate/segment the dopaminergic nuclei, which is necessary for all presented modalities, particularly for those without any specificity for dopaminergic neurons (i.e., morphometric, diffusion, and BOLD MRI). As already described further above, automated segmentation of SNc/VTA is not reliably feasible so far due to insufficient contrast properties of standard MRI techniques. Thus, typical publicly available masks are based on manual delineation of structures in “standard samples” (43, 158, 159). No MRI atlas of dopaminergic sub-nuclei (e.g., substantia nigra subfields) exists so far, as opposed to the cholinergic system (63, 160), for example, where a post-mortem based probabilistic cholinergic basal forebrain delineation in standard brain space is available. Furthermore, accurate delineation requires sufficient spatial resolution. The spatial resolution of the presented MRI techniques is usually not lower than 0.5–1 mm. Given the size of dopaminergic nucleI [VTA ∼60 mm^3^ (161); SN ∼350 mm^3^ (162)], this resolution appears sufficient for a satisfactory delineation of nuclei – still keeping in mind partial volume effects –, but a reliable investigation of sub-nuclei is not possible so far. New techniques like 7T MRI might alleviate these problems in the future, as suggested by promising recent attempts – however, so far, they also rely on manual delineation (163). Furthermore, the multimodal combination with neuromelanin MRI with its good contrast might improve delineation (Figure 6). Finally, instead of using neuromelanin MRI, T1w, or T2w images for delineation, quantitative susceptibility mapping (QSM) could be used for delineation and also morphometric analysis. This novel technique provides enhanced contrast for substantia nigra and VTA by quantifying iron content based on reconstructing magnetic susceptibility sources (164). A recent study manually segmented the substantia nigra based on quantitative susceptibility maps and reported a volume increase in first-episode schizophrenia patients (150).

### Future research questions

First, as already outlined above, integrative multimodal approaches are needed, combining different dopamine MRI methods to investigate complementary aspects of the dopamine systems. This could also improve the delineation of dopaminergic nuclei. Moreover, comparing dopamine MRI methods to dopamine PET methods would help to validate the specificity and reliability of outcomes (Figure 6).

Second, multimodal approaches could be extended to translational approaches, e.g., in rodents, and usage of human post-mortem data. Such studies would strengthen our understanding of the link between dopamine MRI and dopaminergic cellular processes. One example is the study by Cassidy et al., who validated neuromelanin MRI as a proxy measure of dopamine function by combining neuromelanin MRI with neurochemical measurements of neuromelanin concentration in post-mortem tissue of healthy subjects (43). Similar approaches could be conceived for other modalities.

Third, as outlined above, longitudinal approaches in patients with schizophrenia should test whether and how dopamine MRI measures change over time and with changing symptom status, e.g., from psychotic phases to psychotic remission. This would further enhance our understanding of dopaminergic pathology in different phases of schizophrenia and could contribute to the refinement of antidopaminergic medication strategies.

Fourth, schizophrenia is characterized by remarkable heterogeneity, not only at the symptom level, but also on genetic and pathophysiological levels (165, 166). Multimodal approaches on the dopamine system in schizophrenia might help to address the question of heterogenous changes in schizophrenia for the dopamine system, i.e., whether there are subgroups that are particularly affected in the dopamine system or in certain parts of it.

Fifth, there are cases of patients with schizophrenia in whom failures of the dopamine system do not appear to dominate (e.g., patients resistant to antidopaminergic medication). As alternative underpinning pathophysiologies, other disturbed transmitter systems might be relevant, e.g., glutamate or acetylcholine (7). Furthermore, there might be additional pathophysiologies not dominated by transmitter system disturbances, e.g., aberrant neurovascular units (167). Therefore, future studies and reviews should also deal with these questions.

### Conclusion

Although the number of studies is still low, neuromelanin MRI, morphometric MRI, diffusion MRI, and BOLD resting-state fMRI have proven to be viable and complementary methods to investigate the human dopamine system *in vivo* in schizophrenia. We observed a clear trend of results only in neuromelanin MRI – due to a sufficient number of studies and homogeneous outcome measures –, suggesting increased neuromelanin accumulation in the substantia nigra in patients with schizophrenia. A clear trend for gray matter volume changes in VTA/SNc morphometry is not observable so far due to the low number of studies and methodological challenges. In diffusion MRI, there was no clear trend either, largely due to the heterogeneity of outcome measures used across studies. In resting-state BOLD fMRI, there was a weak trend for reduced resting-state functional connectivity of dopaminergic nuclei with forebrain regions, whose interpretation is difficult since the direction of BOLD changes cannot easily be interpreted. As major overarching problems across modalities, we identified most modalities’ lack of specificity for dopaminergic neurons, the low number of studies – particularly systematic investigations of specific patient groups and longitudinal studies are lacking –, and the accurate segmentation of dopaminergic nuclei. Advanced novel MRI techniques and multimodal studies, particularly linking dopamine PET and MRI, might alleviate these problems in the future.

## Author contributions

All authors contributed to conceiving, researching, and writing of this manuscript.

## Abbreviations

BOLD: blood oxygen level-dependent
CNR: contrast-to-noise-ratio
CSD: constrained spherical deconvolution
DTI: diffusion tensor imaging
DWI: diffusion-weighted imaging
FA: fractional anisotropy
FC: functional connectivity
18F-DOPA: 6-18F-fluoro-3,4-dihydoxylphenyl-L-alanine
fMRI: functional magnetic resonance imaging
MNI: Montreal Neurological Institute
MRI: magnetic resonance imaging
PET: positron emission tomography
SNc: substantia nigra pars compacta
VBM: voxel-based morphometry
VTA: ventral tegmental area.

## References

[1] McGrath J Saha S Chant D Welham J. Schizophrenia: a concise overview of incidence, prevalence, and mortality. *Epidemiol Rev.* (2008) 30:67–76. 10.1093/epirev/mxn001 18480098

[2] Chang WC Wong CSM Chen EYH Lam LCW Chan WC Ng RMK Lifetime prevalence and correlates of schizophrenia-spectrum, affective, and other non-affective psychotic disorders in the chinese adult population. *Schizophr Bull.* (2017) 43:1280–90. 10.1093/schbul/sbx056 28586480 PMC5737409

[3] Hjorthøj C Stürup AE McGrath JJ Nordentoft M. Years of potential life lost and life expectancy in schizophrenia: a systematic review and meta-analysis. *Lancet Psychiatry.* (2017) 4:295–301. 10.1016/S2215-0366(17)30078-0 28237639

[4] Bebbington PE Angermeyer M Azorin J-M Brugha T Kilian R Johnson S The European schizophrenia cohort (EuroSC): a naturalistic prognostic and economic study. *Soc Psychiatry Psychiatr Epidemiol.* (2005) 40:707–17. 10.1007/s00127-005-0955-5 16151597

[5] Evensen S Wisløff T Lystad JU Bull H Ueland T Falkum E. Prevalence, employment rate, and cost of schizophrenia in a high-income welfare society: a population-based study using comprehensive health and welfare registers. *Schizophr Bull.* (2016) 42:476–83. 10.1093/schbul/sbv141 26433216 PMC4753607

[6] Howes OD Kapur S. The dopamine hypothesis of schizophrenia: version III–the final common pathway. *Schizophr Bull.* (2009) 35:549–62. 10.1093/schbul/sbp006 19325164 PMC2669582

[7] McCutcheon RA Krystal JH Howes OD. Dopamine and glutamate in schizophrenia: biology, symptoms and treatment. *World Psychiatry.* (2020) 19:15–33. 10.1002/wps.20693 31922684 PMC6953551

[8] Fuxe K. Evidence for the existence of monoamine neurons in the central nervous system. iv. distribution of monoamine nerve terminals in the central nervous system. *Acta Physiol Scand Suppl.* (1965) (Suppl. 247):37. 14319769

[9] Björklund A Dunnett SB. Dopamine neuron systems in the brain: an update. *Trends Neurosci.* (2007) 30:194–202. 10.1016/j.tins.2007.03.006 17408759

[10] Hirsch EC Mouatt A Faucheux B Bonnet AM Javoy-Agid F Graybiel AM Dopamine, tremor, and Parkinson’s disease. *Lancet.* (1992) 340:125–6. 10.1016/0140-6736(92)90457-E 1352004

[11] Kegeles LS Slifstein M Xu X Urban N Thompson JL Moadel T Striatal and extrastriatal dopamine D2/D3 receptors in schizophrenia evaluated with [18F]fallypride positron emission tomography. *Biol Psychiatry.* (2010) 68:634–41. 10.1016/j.biopsych.2010.05.027 20673873 PMC2952433

[12] Weinstein JJ Chohan MO Slifstein M Kegeles LS Moore H Abi-Dargham A. Pathway-specific dopamine abnormalities in schizophrenia. *Biol Psychiatry.* (2017) 81:31–42. 10.1016/j.biopsych.2016.03.2104 27206569 PMC5177794

[13] Tritsch NX Sabatini BL. Dopaminergic modulation of synaptic transmission in cortex and striatum. *Neuron.* (2012) 76:33–50. 10.1016/j.neuron.2012.09.023 23040805 PMC4386589

[14] Uchigashima M Ohtsuka T Kobayashi K Watanabe M. Dopamine synapse is a neuroligin-2-mediated contact between dopaminergic presynaptic and GABAergic postsynaptic structures. *Proc Natl Acad Sci USA.* (2016) 113:4206–11. 10.1073/pnas.1514074113 27035941 PMC4839454

[15] Peroutka SJ Synder SH. Relationship of neuroleptic drug effects at brain dopamine, serotonin, alpha-adrenergic, and histamine receptors to clinical potency. *Am J Psychiatry.* (1980) 137:1518–22. 10.1176/ajp.137.12.1518 6108081

[16] Howes OD Kambeitz J Kim E Stahl D Slifstein M Abi-Dargham A The nature of dopamine dysfunction in schizophrenia and what this means for treatment. *Arch Gen Psychiatry.* (2012) 69:776–86. 10.1001/archgenpsychiatry.2012.169 22474070 PMC3730746

[17] Fusar-Poli P Meyer-Lindenberg A. Striatal presynaptic dopamine in schizophrenia, part II: meta-analysis of [(18)F/(11)C]-DOPA PET studies. *Schizophr Bull.* (2013) 39:33–42. 10.1093/schbul/sbr180 22282454 PMC3523905

[18] McCutcheon R Beck K Jauhar S Howes OD. Defining the locus of dopaminergic dysfunction in schizophrenia: a meta-analysis and test of the mesolimbic hypothesis. *Schizophr Bull.* (2018) 44:1301–11. 10.1093/schbul/sbx180 29301039 PMC5933516

[19] Avram M Brandl F Cabello J Leucht C Scherr M Mustafa M Reduced striatal dopamine synthesis capacity in patients with schizophrenia during remission of positive symptoms. *Brain.* (2019) 142:1813–26. 31135051 10.1093/brain/awz093

[20] Kim E Howes OD Veronese M Beck K Seo S Park JW Presynaptic dopamine capacity in patients with treatment-resistant schizophrenia taking clozapine: an [18F]DOPA PET study. *Neuropsychopharmacology.* (2017) 42:941–50. 10.1038/npp.2016.258 27857125 PMC5312074

[21] Kim S Shin SH Santangelo B Veronese M Kang SK Lee JS Dopamine dysregulation in psychotic relapse after antipsychotic discontinuation: an [18F]DOPA and [11C]raclopride PET study in first-episode psychosis. *Mol Psychiatry.* (2021) 26:3476–88. 10.1038/s41380-020-00879-0 32929214

[22] Fusar-Poli P Meyer-Lindenberg A. Striatal presynaptic dopamine in schizophrenia, part I: meta-analysis of dopamine active transporter (DAT) density. *Schizophr Bull.* (2013) 39:22–32. 10.1093/schbul/sbr111 22282456 PMC3523907

[23] Kambeitz J Abi-Dargham A Kapur S Howes OD. Alterations in cortical and extrastriatal subcortical dopamine function in schizophrenia: systematic review and meta-analysis of imaging studies. *Br J Psychiatry.* (2014) 204:420–9. 10.1192/bjp.bp.113.132308 25029687

[24] Brugger SP Angelescu I Abi-Dargham A Mizrahi R Shahrezaei V Howes OD. Heterogeneity of striatal dopamine function in schizophrenia: meta-analysis of variance. *Biol Psychiatry.* (2020) 87:215–24. 10.1016/j.biopsych.2019.07.008 31561858

[25] Egerton A Demjaha A McGuire P Mehta MA Howes OD. The test-retest reliability of 18F-DOPA PET in assessing striatal and extrastriatal presynaptic dopaminergic function. *Neuroimage.* (2010) 50:524–31. 10.1016/j.neuroimage.2009.12.058 20034580 PMC4096947

[26] Howes OD Williams M Ibrahim K Leung G Egerton A McGuire PK Midbrain dopamine function in schizophrenia and depression: a post-mortem and positron emission tomographic imaging study. *Brain.* (2013) 136:3242–51. 10.1093/brain/awt264 24097339 PMC3808688

[27] Kumakura Y Cumming P Vernaleken I Buchholz H-G Siessmeier T Heinz A Elevated [18F]fluorodopamine turnover in brain of patients with schizophrenia: an [18F]fluorodopa/positron emission tomography study. *J Neurosci.* (2007) 27:8080–7. 10.1523/JNEUROSCI.0805-07.2007 17652599 PMC6672729

[28] Elkashef AM Doudet D Bryant T Cohen RM Li S-H Wyatt RJ. 6-18F-DOPA PET study in patients with schizophrenia. *Psychiatry Res Neuroimaging.* (2000) 100:1–11. 10.1016/s0925-4927(00)00064-0 11090720

[29] Kumakura Y Cumming P. PET studies of cerebral levodopa metabolism: a review of clinical findings and modeling approaches. *Neuroscientist.* (2009) 15:635–50. 10.1177/1073858409338217 19793723

[30] Horga G Wengler K Cassidy CM. Neuromelanin-sensitive magnetic resonance imaging as a proxy marker for catecholamine function in psychiatry. *JAMA Psychiatry.* (2021) 78:788–9. 10.1001/jamapsychiatry.2021.0927 34009285 PMC9060608

[31] Swartz HM Sarna T Zecca L. Modulation by neuromelanin of the availability and reactivity of metal ions. *Ann Neurol.* (1992) 32:S69–75. 10.1002/ana.410320712 1510383

[32] Zecca L Bellei C Costi P Albertini A Monzani E Casella L New melanic pigments in the human brain that accumulate in aging and block environmental toxic metals. *Proc Natl Acad Sci USA.* (2008) 105:17567–72. 10.1073/pnas.0808768105 18988735 PMC2582310

[33] Zucca FA Vanna R Cupaioli FA Bellei C De Palma A Di Silvestre D Neuromelanin organelles are specialized autolysosomes that accumulate undegraded proteins and lipids in aging human brain and are likely involved in Parkinson’s disease. *npj Parkinson’s Dis.* (2018) 4:1–23. 10.1038/s41531-018-0050-8 29900402 PMC5988730

[34] Zecca L Swartz HM. Total and paramagnetic metals in human substantia nigra and its neuromelanin. *J Neural Transm Park Dis Dement Sect.* (1993) 5:203–13. 10.1007/BF02257675 8396395

[35] Sulzer D Cassidy C Horga G Kang UJ Fahn S Casella L Neuromelanin detection by magnetic resonance imaging (MRI) and its promise as a biomarker for Parkinson’s disease. *NPJ Parkinsons Dis.* (2018) 4:11. 10.1038/s41531-018-0047-3 29644335 PMC5893576

[36] Tanttu JI Sepponen RE Lipton MJ Kuusela T. Synergistic enhancement of MRI with Gd-DTPA and magnetization transfer. *J Comput Assist Tomogr.* (1992) 16:19–24. 10.1097/00004728-199201000-00004 1729300

[37] Tosk JM Holshouser BA Aloia RC Hinshaw DB Jr. Hasso AN MacMurray JP Effects of the interaction between ferric iron and L-dopa melanin on T1 and T2 relaxation times determined by magnetic resonance imaging. *Magn Reson Med.* (1992) 26:40–5. 10.1002/mrm.1910260105 1625565

[38] Trujillo P Summers PE Ferrari E Zucca FA Sturini M Mainardi LT Contrast mechanisms associated with neuromelanin-MRI. *Magn Reson Med.* (2017) 78:1790–800. 10.1002/mrm.26584 28019018

[39] Sasaki M Shibata E Tohyama K Takahashi J Otsuka K Tsuchiya K Neuromelanin magnetic resonance imaging of locus ceruleus and substantia nigra in Parkinson’s disease. *Neuroreport.* (2006) 17:1215–8. 10.1097/01.wnr.0000227984.84927.a7 16837857

[40] Wieland L Fromm S Hetzer S Schlagenhauf F Kaminski J. Neuromelanin-sensitive magnetic resonance imaging in schizophrenia: a meta-analysis of case-control studies. *Front Psychiatry.* (2021) 12:770282. 10.3389/fpsyt.2021.770282 34777070 PMC8581671

[41] Cho SJ Bae YJ Kim J-M Kim D Baik SH Sunwoo L Diagnostic performance of neuromelanin-sensitive magnetic resonance imaging for patients with Parkinson’s disease and factor analysis for its heterogeneity: a systematic review and meta-analysis. *Eur Radiol.* (2021) 31:1268–80. 10.1007/s00330-020-07240-7 32886201

[42] Wang X Zhang Y Zhu C Li G Kang J Chen F The diagnostic value of SNpc using NM-MRI in Parkinson’s disease: meta-analysis. *Neurol Sci.* (2019) 40:2479–89. 10.1007/s10072-019-04014-y 31392640

[43] Cassidy CM Zucca FA Girgis RR Baker SC Weinstein JJ Sharp ME Neuromelanin-sensitive MRI as a noninvasive proxy measure of dopamine function in the human brain. *Proc Natl Acad Sci USA.* (2019) 116:5108–17. 10.1073/pnas.1807983116 30796187 PMC6421437

[44] Hirsch E Graybiel AM Agid YA. Melanized dopaminergic neurons are differentially susceptible to degeneration in Parkinson’s disease. *Nature.* (1988) 334:345–8. 10.1038/334345a0 2899295

[45] Wengler K He X Abi-Dargham A Horga G. Reproducibility assessment of neuromelanin-sensitive magnetic resonance imaging protocols for region-of-interest and voxelwise analyses. *Neuroimage.* (2020) 208:116457. 10.1016/j.neuroimage.2019.116457 31841683 PMC7118586

[46] Shibata E Sasaki M Tohyama K Otsuka K Endoh J Terayama Y Use of neuromelanin-sensitive MRI to distinguish schizophrenic and depressive patients and healthy individuals based on signal alterations in the substantia nigra and locus ceruleus. *Biol Psychiatry.* (2008) 64:401–6. 10.1016/j.biopsych.2008.03.021 18452894

[47] Ueno F Iwata Y Nakajima S Caravaggio F Rubio JM Horga G Neuromelanin accumulation in patients with schizophrenia: a systematic review and meta-analysis. *Neurosci Biobehav Rev.* (2022) 132:1205–13. 10.1016/j.neubiorev.2021.10.028 34718049 PMC9059704

[48] Sasaki M Shibata E Ohtsuka K Endoh J Kudo K Narumi S Visual discrimination among patients with depression and schizophrenia and healthy individuals using semiquantitative color-coded fast spin-echo T1-weighted magnetic resonance imaging. *Neuroradiology.* (2010) 52:83–9. 10.1007/s00234-009-0595-7 19756561

[49] Yamashita F Sasaki M Fukumoto K Otsuka K Uwano I Kameda H Detection of changes in the ventral tegmental area of patients with schizophrenia using neuromelanin-sensitive MRI. *Neuroreport.* (2016) 27:289–94. 10.1097/WNR.0000000000000530 26901057

[50] Cassidy CM Carpenter KM Konova AB Cheung V Grassetti A Zecca L Evidence for dopamine abnormalities in the substantia nigra in cocaine addiction revealed by neuromelanin-sensitive MRI. *Am J Psychiatry.* (2020) 177:1038–47. 10.1176/appi.ajp.2020.20010090 32854531 PMC9108998

[51] Jalles C Chendo I Levy P Reimão S. Neuromelanin changes in first episode psychosis with substance abuse. *Schizophr Res.* (2020) 220:283–4. 10.1016/j.schres.2020.03.034 32253077

[52] Wengler K Ashinoff BK Pueraro E Cassidy CM Horga G Rutherford BR. Association between neuromelanin-sensitive MRI signal and psychomotor slowing in late-life depression. *Neuropsychopharmacology.* (2021) 46:1233–9. 10.1038/s41386-020-00860-z 32919398 PMC8134510

[53] Berger A. Magnetic resonance imaging. *BMJ.* (2002) 324:35. 10.1136/bmj.324.7328.35 11777806 PMC1121941

[54] Backhausen LL Herting MM Tamnes CK Vetter NC. Best practices in structural neuroimaging of neurodevelopmental disorders. *Neuropsychol Rev.* (2021) 32:400–18. 10.1007/s11065-021-09496-2 33893904 PMC9090677

[55] Ashburner J Friston KJ. Voxel-based morphometry–the methods. *Neuroimage.* (2000) 11:805–21. 10.1006/nimg.2000.0582 10860804

[56] Ashburner J Friston KJ. Why voxel-based morphometry should be used. *Neuroimage.* (2001) 14:1238–43. 10.1006/nimg.2001.0961 11707080

[57] Good CD Johnsrude IS Ashburner J Henson RN Friston KJ Frackowiak RS. A voxel-based morphometric study of ageing in 465 normal adult human brains. *Neuroimage.* (2001) 14:21–36. 10.1006/nimg.2001.0786 11525331

[58] Fischl B Salat DH Busa E Albert M Dieterich M Haselgrove C Whole brain segmentation: neurotechnique automated labeling of neuroanatomical structures in the human brain. *Neuron.* (2002) 33:341–55. 10.1016/s0896-6273(02)00569-x 11832223

[59] Fischl B Salat DH van der Kouwe AJW Makris N Ségonne F Quinn BT Sequence-independent segmentation of magnetic resonance images. *Neuroimage.* (2004) 23(Suppl. 1):S69–84. 10.1016/j.neuroimage.2004.07.016 15501102

[60] Dzieciol K Iordanishvili E Abbas Z Nahimi A Winterdahl M Shah NJ. A robust method for the detection of small changes in relaxation parameters and free water content in the vicinity of the substantia nigra in Parkinson’s disease patients. *PLoS One.* (2021) 16:e0247552. 10.1371/journal.pone.0247552 33626092 PMC7904163

[61] Feraco P Gagliardo C La Tona G Bruno E D’angelo C Marrale M Imaging of substantia nigra in Parkinson’s disease: a narrative review. *Brain Sci.* (2021) 11:769. 10.3390/brainsci11060769 34207681 PMC8230134

[62] Helms G Draganski B Frackowiak R Ashburner J Weiskopf N. Improved segmentation of deep brain grey matter structures using magnetization transfer (MT) parameter maps. *Neuroimage.* (2009) 47:194–8. 10.1016/j.neuroimage.2009.03.053 19344771 PMC2694257

[63] Grothe MJ Scheef L Bäuml J Meng C Daamen M Baumann N Reduced cholinergic basal forebrain integrity links neonatal complications and adult cognitive deficits after premature birth. *Biol Psychiatry.* (2017) 82:119–26. 10.1016/j.biopsych.2016.12.008 28129944

[64] Avram M Grothe MJ Meinhold L Leucht C Leucht S Borgwardt S Lower cholinergic basal forebrain volumes link with cognitive difficulties in schizophrenia. *Neuropsychopharmacology.* (2021) 46:2320–9. 10.1038/s41386-021-01070-x 34188186 PMC8580980

[65] Visser E Keuken MC Douaud G Gaura V Bachoud-Levi A-C Remy P Automatic segmentation of the striatum and globus pallidus using MIST: multimodal image segmentation tool. *Neuroimage.* (2016) 125:479–97. 10.1016/j.neuroimage.2015.10.013 26477650 PMC4692519

[66] Manjón JV Bertó A Romero JE Lanuza E Vivo-Hernando R Aparici-Robles F pBrain: a novel pipeline for Parkinson related brain structure segmentation. *NeuroImage Clin.* (2020) 25:102184. 10.1016/j.nicl.2020.102184 31982678 PMC6992999

[67] Weinberger DR Radulescu E. Finding the elusive psychiatric “lesion” With 21st-Century neuroanatomy: a note of caution. *AJP.* (2016) 173:27–33. 10.1176/appi.ajp.2015.15060753 26315983

[68] Ge Q Peng W Zhang J Weng X Zhang Y Liu T Short-term apparent brain tissue changes are contributed by cerebral blood flow alterations. *PLoS One.* (2017) 12:e0182182. 10.1371/journal.pone.0182182 28820894 PMC5562307

[69] Gupta CN Calhoun VD Rachakonda S Chen J Patel V Liu J Patterns of gray matter abnormalities in schizophrenia based on an international mega-analysis. *Schizophr Bull.* (2015) 41:1133–42. 10.1093/schbul/sbu177 25548384 PMC4535628

[70] Ellison-Wright I Bullmore E. Anatomy of bipolar disorder and schizophrenia: a meta-analysis. *Schizophr Res.* (2010) 117:1–12. 10.1016/j.schres.2009.12.022 20071149

[71] Brandl F Avram M Weise B Shang J Simões B Bertram T Specific substantial dysconnectivity in schizophrenia: a transdiagnostic multimodal meta-analysis of resting-state functional and structural magnetic resonance imaging studies. *Biol Psychiatry.* (2019) 85:573–83. 10.1016/j.biopsych.2018.12.003 30691673

[72] Kempton MJ Haldane M Jogia J Grasby PM Collier D Frangou S. Dissociable brain structural changes associated with predisposition, resilience, and disease expression in bipolar disorder. *J Neurosci.* (2009) 29:10863–8. 10.1523/JNEUROSCI.2204-09.2009 19726644 PMC6665540

[73] Shad MU Muddasani S Rao U. Gray matter differences between healthy and depressed adolescents: a voxel-based morphometry study. *J Child Adolesc Psychopharmacol.* (2012) 22:190–7. 10.1089/cap.2011.0005 22537357 PMC3373217

[74] Pando-Naude V Toxto S Fernandez-Lozano S Parsons CE Alcauter S Garza-Villarreal EA. Gray and white matter morphology in substance use disorders: a neuroimaging systematic review and meta-analysis. *Transl Psychiatry.* (2021) 11:29. 10.1038/s41398-020-01128-2 33431833 PMC7801701

[75] Wise T Radua J Via E Cardoner N Abe O Adams TM Common and distinct patterns of grey-matter volume alteration in major depression and bipolar disorder: evidence from voxel-based meta-analysis. *Mol Psychiatry.* (2017) 22:1455–63. 10.1038/mp.2016.72 27217146 PMC5622121

[76] Brandl F Weise B Mulej Bratec S Jassim N Hoffmann Ayala D Bertram T Common and specific large-scale brain changes in major depressive disorder, anxiety disorders, and chronic pain: a transdiagnostic multimodal meta-analysis of structural and functional MRI studies. *Neuropsychopharmacology.* (2022) 47:1071–80. 10.1038/s41386-022-01271-y 35058584 PMC8938548

[77] Geng D-Y Li Y-X Zee C-S. Magnetic resonance imaging-based volumetric analysis of basal ganglia nuclei and substantia nigra in patients with Parkinson’s disease. *Neurosurgery.* (2006) 58:256–62;discussion256–62. 10.1227/01.NEU.0000194845.19462.7B 16462479

[78] Sako W Murakami N Izumi Y Kaji R. MRI can detect nigral volume loss in patients with Parkinson’s disease: evidence from a meta-analysis. *J Parkinsons Dis.* (2014) 4:405–11. 10.3233/JPD-130332 24577503

[79] Le Bihan D Breton E. *In vivo* magnetic resonance imaging of diffusion. *Compt Rend Seanc Acad Sci Ser.* (1985) 301:1109–12.

[80] Basser PJ. Inferring microstructural features and the physiological state of tissues from diffusion-weighted images. *NMR Biomed.* (1995) 8:333–44. 10.1002/nbm.1940080707 8739270

[81] Le Bihan D. Looking into the functional architecture of the brain with diffusion MRI. *Nat Rev Neurosci.* (2003) 4:469–80. 10.1038/nrn1119 12778119

[82] Moseley ME Cohen Y Kucharczyk J Mintorovitch J Asgari HS Wendland MF Diffusion-weighted MR imaging of anisotropic water diffusion in cat central nervous system. *Radiology.* (1990) 176:439–45. 10.1148/radiology.176.2.2367658 2367658

[83] Pierpaoli C Basser PJ. Toward a quantitative assessment of diffusion anisotropy. *Magn Reson Med.* (1996) 36:893–906. 10.1002/mrm.1910360612 8946355

[84] Jellison BJ Field AS Medow J Lazar M Salamat MS Alexander AL. Diffusion tensor imaging of cerebral white matter: a pictorial review of physics, fiber tract anatomy, and tumor imaging patterns. *AJNR Am J Neuroradiol.* (2004) 25:356–69. 15037456 PMC8158568

[85] Basser PJ Pierpaoli C. Microstructural and physiological features of tissues elucidated by quantitative-diffusion-tensor MRI. *J Magn Reson B.* (1996) 111:209–19. 10.1006/jmrb.1996.0086 8661285

[86] Soares JM Marques P Alves V Sousa N. A hitchhiker’s guide to diffusion tensor imaging. *Front Neurosci.* (2013) 7:31. 10.3389/fnins.2013.00031 23486659 PMC3594764

[87] Rheault F Houde J-C Descoteaux M. Visualization, interaction and tractometry: dealing with millions of streamlines from diffusion MRI tractography. *Front Neuroinform.* (2017) 11:42. 10.3389/fninf.2017.00042 28694776 PMC5483435

[88] Mori S Crain BJ Chacko VP van Zijl PC. Three-dimensional tracking of axonal projections in the brain by magnetic resonance imaging. *Ann Neurol.* (1999) 45:265–9. 10.1002/1531-8249(199902)45:2<265::AID-ANA21>3.0.CO;2-3 9989633

[89] Behrens TEJ Woolrich MW Jenkinson M Johansen-Berg H Nunes RG Clare S Characterization and propagation of uncertainty in diffusion-weighted MR imaging. *Magn Reson Med.* (2003) 50:1077–88. 10.1002/mrm.10609 14587019

[90] Behrens TEJ Johansen-Berg H Woolrich MW Smith SM Wheeler-Kingshott CAM Boulby PA Non-invasive mapping of connections between human thalamus and cortex using diffusion imaging. *Nat Neurosci.* (2003) 6:750–7. 10.1038/nn1075 12808459

[91] Zhang Y Larcher KM-H Misic B Dagher A. Anatomical and functional organization of the human substantia nigra and its connections. *Elife.* (2017) 6:e26653. 10.7554/eLife.26653 28826495 PMC5606848

[92] Basile GA Bramanti A Bertino S Cutroneo G Bruno A Tisano A Structural connectivity-based parcellation of the dopaminergic midbrain in healthy subjects and schizophrenic patients. *Medicina.* (2020) 56:686. 10.3390/medicina56120686 33322072 PMC7764101

[93] Bracht T Horn H Strik W Federspiel A Razavi N Stegmayer K White matter pathway organization of the reward system is related to positive and negative symptoms in schizophrenia. *Schizophr Res.* (2014) 153:136–42. 10.1016/j.schres.2014.01.015 24485586

[94] Vosberg DE Zhang Y Menegaux A Chalupa A Manitt C Zehntner S Mesocorticolimbic connectivity and volumetric alterations in DCC mutation carriers. *J Neurosci.* (2018) 38:4655–65. 10.1523/JNEUROSCI.3251-17.2018 29712788 PMC5956985

[95] Jbabdi S Johansen-Berg H. Tractography: where do we go from here? *Brain Connect.* (2011) 1:169–83. 10.1089/brain.2011.0033 22433046 PMC3677805

[96] MacKay A Laule C Vavasour I Bjarnason T Kolind S Mädler B. Insights into brain microstructure from the T2 distribution. *Magn Reson Imaging.* (2006) 24:515–25. 10.1016/j.mri.2005.12.037 16677958

[97] Zhang H Schneider T Wheeler-Kingshott CA Alexander DC. NODDI: practical *in vivo* neurite orientation dispersion and density imaging of the human brain. *Neuroimage.* (2012) 61:1000–16. 10.1016/j.neuroimage.2012.03.072 22484410

[98] Jones DK Knösche TR Turner R. White matter integrity, fiber count, and other fallacies: the do’s and don’ts of diffusion MRI. *Neuroimage.* (2013) 73:239–54. 10.1016/j.neuroimage.2012.06.081 22846632

[99] Basser PJ Pajevic S Pierpaoli C Duda J Aldroubi A. *In vivo* fiber tractography using DT-MRI data. *Magn Reson Med.* (2000) 44:625–32. 10.1002/1522-2594(200010)44:4<625::AID-MRM17>3.0.CO;2-O 11025519

[100] Alexander DC Barker GJ Arridge SR. Detection and modeling of non-Gaussian apparent diffusion coefficient profiles in human brain data. *Magn Reson Med.* (2002) 48:331–40. 10.1002/mrm.10209 12210942

[101] Tuch DS Reese TG Wiegell MR Makris N Belliveau JW Wedeen VJ. High angular resolution diffusion imaging reveals intravoxel white matter fiber heterogeneity. *Magn Reson Med.* (2002) 48:577–82. 10.1002/mrm.10268 12353272

[102] Tournier J-D Calamante F Gadian DG Connelly A. Direct estimation of the fiber orientation density function from diffusion-weighted MRI data using spherical deconvolution. *Neuroimage.* (2004) 23:1176–85. 10.1016/j.neuroimage.2004.07.037 15528117

[103] Bracht T Viher PV Stegmayer K Strik W Federspiel A Wiest R Increased structural connectivity of the medial forebrain bundle in schizophrenia spectrum disorders is associated with delusions of paranoid threat and grandiosity. *Neuroimage Clin.* (2019) 24:102044. 10.1016/j.nicl.2019.102044 31678911 PMC6978276

[104] Giordano GM Pezzella P Quarantelli M Bucci P Prinster A Soricelli A Investigating the relationship between white matter connectivity and motivational circuits in subjects with deficit schizophrenia: a diffusion tensor imaging (DTI) study. *J Clin Med Res.* (2021) 11:61. 10.3390/jcm11010061 35011803 PMC8745695

[105] Savadjiev P Rathi Y Bouix S Smith AR Schultz RT Verma R Fusion of white and gray matter geometry: a framework for investigating brain development. *Med Image Anal.* (2014) 18:1349–60. 10.1016/j.media.2014.06.013 25066750 PMC4162846

[106] Rivas-Grajales AM Savadjiev P Kubicki M Nestor PG Niznikiewicz M McCarley RW Striato-nigro-striatal tract dispersion abnormalities in patients with chronic schizophrenia. *Brain Imaging Behav.* (2019) 13:1236–45. 10.1007/s11682-018-9934-9 30109597

[107] Kwon HG Jang SH. Differences in neural connectivity between the substantia nigra and ventral tegmental area in the human brain. *Front Hum Neurosci.* (2014) 8:41. 10.3389/fnhum.2014.00041 24567711 PMC3915097

[108] Chowdhury R Lambert C Dolan RJ Düzel E. Parcellation of the human substantia nigra based on anatomical connectivity to the striatum. *Neuroimage.* (2013) 81:191–8. 10.1016/j.neuroimage.2013.05.043 23684858 PMC3734352

[109] Coenen VA Honey CR Hurwitz T Rahman AA McMaster J Bürgel U Medial forebrain bundle stimulation as a pathophysiological mechanism for hypomania in subthalamic nucleus deep brain stimulation for Parkinson’s disease. *Neurosurgery.* (2009) 64:1106–14;discussion1114–5. 10.1227/01.NEU.0000345631.54446.06 19487890

[110] Coenen VA Panksepp J Hurwitz TA Urbach H Mädler B. Human medial forebrain bundle (MFB) and anterior thalamic radiation (ATR): imaging of two major subcortical pathways and the dynamic balance of opposite affects in understanding depression. *J Neuropsychiatry Clin Neurosci.* (2012) 24:223–36. 10.1176/appi.neuropsych.11080180 22772671

[111] Bracht T Doidge AN Keedwell PA Jones DK. Hedonic tone is associated with left supero-lateral medial forebrain bundle microstructure. *Psychol Med.* (2015) 45:865–74. 10.1017/S0033291714001949 25124530 PMC4413785

[112] Coenen VA Schumacher LV Kaller C Schlaepfer TE Reinacher PC Egger K The anatomy of the human medial forebrain bundle: ventral tegmental area connections to reward-associated subcortical and frontal lobe regions. *Neuroimage Clin.* (2018) 18:770–83. 10.1016/j.nicl.2018.03.019 29845013 PMC5964495

[113] Denier N Walther S Schneider C Federspiel A Wiest R Bracht T. Reduced tract length of the medial forebrain bundle and the anterior thalamic radiation in bipolar disorder with melancholic depression. *J Affect Disord.* (2020) 274:8–14. 10.1016/j.jad.2020.05.008 32469836

[114] MacNiven KH Leong JK Knutson B. Medial forebrain bundle structure is linked to human impulsivity. *Sci Adv.* (2020) 6:eaba4788. 10.1126/sciadv.aba4788 32938676 PMC7494337

[115] Ashtari M Cervellione K Cottone J Ardekani BA Sevy S Kumra S. Diffusion abnormalities in adolescents and young adults with a history of heavy cannabis use. *J Psychiatr Res.* (2009) 43:189–204. 10.1016/j.jpsychires.2008.12.002 19111160 PMC3314332

[116] Hampton WH Hanik IM Olson IR. Substance abuse and white matter: findings, limitations, and future of diffusion tensor imaging research. *Drug Alcohol Depend.* (2019) 197:288–98. 10.1016/j.drugalcdep.2019.02.005 30875650 PMC6440853

[117] Yoshikawa K Nakata Y Yamada K Nakagawa M. Early pathological changes in the parkinsonian brain demonstrated by diffusion tensor MRI. *J Neurol Neurosurg Psychiatry.* (2004) 75:481–4. 10.1136/jnnp.2003.021873 14966170 PMC1738942

[118] Menke RA Scholz J Miller KL Deoni S Jbabdi S Matthews PM MRI characteristics of the substantia nigra in Parkinson’s disease: a combined quantitative T1 and DTI study. *Neuroimage.* (2009) 47:435–41. 10.1016/j.neuroimage.2009.05.017 19447183

[119] Lehéricy S Sharman MA Dos Santos CL Paquin R Gallea C. Magnetic resonance imaging of the substantia nigra in Parkinson’s disease. *Mov Disord.* (2012) 27:822–30. 10.1002/mds.25015 22649063

[120] Ogawa S Lee TM Kay AR Tank DW. Brain magnetic resonance imaging with contrast dependent on blood oxygenation. *Proc Natl Acad Sci USA.* (1990) 87:9868–72. 10.1073/pnas.87.24.9868 2124706 PMC55275

[121] Heeger DJ Ress D. What does fMRI tell us about neuronal activity? *Nat Rev Neurosci.* (2002) 3:142–51. 10.1038/nrn730 11836522

[122] Buxton RB. The physics of functional magnetic resonance imaging (fMRI). *Rep Prog Phys.* (2013) 76:096601. 10.1088/0034-4885/76/9/096601 PMC4376284 24006360

[123] Drew PJ Mateo C Turner KL Yu X Kleinfeld D. Ultra-slow Oscillations in fMRI and resting-state connectivity: neuronal and vascular contributions and technical confounds. *Neuron.* (2020) 107:782–804. 10.1016/j.neuron.2020.07.020 32791040 PMC7886622

[124] Fox MD Raichle ME. Spontaneous fluctuations in brain activity observed with functional magnetic resonance imaging. *Nat Rev Neurosci.* (2007) 8:700–11. 10.1038/nrn2201 17704812

[125] Biswal B Yetkin FZ Haughton VM Hyde JS. Functional connectivity in the motor cortex of resting human brain using echo-planar MRI. *Magn Reson Med.* (1995) 34:537–41. 10.1002/mrm.1910340409 8524021

[126] Biswal BB Mennes M Zuo X-N Gohel S Kelly C Smith SM Toward discovery science of human brain function. *Proc Natl Acad Sci USA.* (2010) 107:4734–9. 10.1073/pnas.0911855107 20176931 PMC2842060

[127] Buckner RL Krienen FM Yeo BTT. Opportunities and limitations of intrinsic functional connectivity MRI. *Nat Neurosci.* (2013) 16:832–7. 10.1038/nn.3423 23799476

[128] Düzel E Bunzeck N Guitart-Masip M Wittmann B Schott BH Tobler PN. Functional imaging of the human dopaminergic midbrain. *Trends Neurosci.* (2009) 32:321–8. 10.1016/j.tins.2009.02.005 19446348

[129] Woodward ND Cascio CJ. Resting-state functional connectivity in psychiatric disorders. *JAMA Psychiatry.* (2015) 72:743–4. 10.1001/jamapsychiatry.2015.0484 26061674 PMC4693599

[130] Haber SN Fudge JL. The primate substantia nigra and VTA: integrative circuitry and function. *Crit Rev Neurobiol.* (1997) 11:323–42. 10.1615/CritRevNeurobiol.v11.i4.40 9336716

[131] Morales M Margolis EB. Ventral tegmental area: cellular heterogeneity, connectivity and behaviour. *Nat Rev Neurosci.* (2017) 18:73–85. 10.1038/nrn.2016.165 28053327

[132] García-Cabezas MA Martínez-Sánchez P Sánchez-González MA Garzón M Cavada C. Dopamine innervation in the thalamus: monkey versus rat. *Cereb Cortex.* (2009) 19:424–34. 10.1093/cercor/bhn093 18550594 PMC2638784

[133] Kelly C de Zubicaray G Di Martino A Copland DA Reiss PT Klein DF L-dopa modulates functional connectivity in striatal cognitive and motor networks: a double-blind placebo-controlled study. *J Neurosci.* (2009) 29:7364–78. 10.1523/JNEUROSCI.0810-09.2009 19494158 PMC2928147

[134] Cole DM Oei NYL Soeter RP Both S van Gerven JMA Rombouts SARB Dopamine-dependent architecture of cortico-subcortical network connectivity. *Cereb Cortex.* (2013) 3:1509–16. 10.1093/cercor/bhs136 22645252

[135] Kohno M Okita K Morales AM Robertson CL Dean AC Ghahremani DG Midbrain functional connectivity and ventral striatal dopamine D2-type receptors: link to impulsivity in methamphetamine users. *Mol Psychiatry.* (2016) 21:1554–60. 10.1038/mp.2015.223 26830141 PMC4970974

[136] Shafiei G Zeighami Y Clark CA Coull JT Nagano-Saito A Leyton M Dopamine signaling modulates the stability and integration of intrinsic brain networks. *Cereb Cortex.* (2019) 29:397–409. 10.1093/cercor/bhy264 30357316 PMC6294404

[137] Liu TT. Noise contributions to the fMRI signal: an overview. *Neuroimage.* (2016) 143:141–51. 10.1016/j.neuroimage.2016.09.008 27612646

[138] Greitz D Franck A Nordell B. On the pulsatile nature of intracranial and spinal CSF-circulation demonstrated by MR imaging. *Acta Radiol.* (1993) 34:321–8. 10.1080/02841859309173251 8318291

[139] Dagli MS Ingeholm JE Haxby JV. Localization of cardiac-induced signal change in fMRI. *Neuroimage.* (1999) 9:407–15. 10.1006/nimg.1998.0424 10191169

[140] Brooks JCW Faull OK Pattinson KTS Jenkinson M. Physiological noise in brainstem FMRI. *Front Hum Neurosci.* (2013) 7:623. 10.3389/fnhum.2013.00623 24109446 PMC3790256

[141] Beissner F. Functional MRI of the brainstem: common problems and their solutions. *Clin Neuroradiol.* (2015) 25(Suppl. 2):251–7. 10.1007/s00062-015-0404-0 25981409

[142] Katyal S Greene CA Ress D. High-resolution functional magnetic resonance imaging methods for human midbrain. *J Vis Exp.*(2012) 63:e3746. 10.3791/3746 22617680 PMC3468195

[143] Deistung A Schäfer A Schweser F Biedermann U Güllmar D Trampel R High-resolution MR imaging of the human brainstem *in vivo* at 7 tesla. *Front Hum Neurosci.* (2013) 7:710. 10.3389/fnhum.2013.00710 24194710 PMC3810670

[144] Caballero-Gaudes C Reynolds RC. Methods for cleaning the BOLD fMRI signal. *Neuroimage.* (2017) 154:128–49. 10.1016/j.neuroimage.2016.12.018 27956209 PMC5466511

[145] Hadley JA Nenert R Kraguljac NV Bolding MS White DM Skidmore FM Ventral tegmental area/midbrain functional connectivity and response to antipsychotic medication in schizophrenia. *Neuropsychopharmacology.* (2014) 39:1020–30. 10.1038/npp.2013.305 24165885 PMC3924537

[146] Giordano GM Stanziano M Papa M Mucci A Prinster A Soricelli A Functional connectivity of the ventral tegmental area and avolition in subjects with schizophrenia: a resting state functional MRI study. *Eur Neuropsychopharmacol.* (2018) 28:589–602. 10.1016/j.euroneuro.2018.03.013 29653743

[147] Xu P Klaasen NG Opmeer EM Pijnenborg GHM van Tol M-J Liemburg EJ Intrinsic mesocorticolimbic connectivity is negatively associated with social amotivation in people with schizophrenia. *Schizophr Res.* (2019) 208:353–9. 10.1016/j.schres.2019.01.023 30711314

[148] Gu H Salmeron BJ Ross TJ Geng X Zhan W Stein EA Mesocorticolimbic circuits are impaired in chronic cocaine users as demonstrated by resting-state functional connectivity. *Neuroimage.* (2010) 53:593–601. 10.1016/j.neuroimage.2010.06.066 20603217 PMC2930044

[149] Nakamura Y Okada N Koshiyama D Kamiya K Abe O Kunimatsu A Differences in functional connectivity networks related to the midbrain dopaminergic system-related area in various psychiatric disorders. *Schizophr Bull.* (2020) 46:1239–48. 10.1093/schbul/sbz121 31901932 PMC7505191

[150] Xu M Guo Y Cheng J Xue K Yang M Song X Brain iron assessment in patients with first-episode schizophrenia using quantitative susceptibility mapping. *NeuroImage Clin.* (2021) 31:102736. 10.1016/j.nicl.2021.102736 34186296 PMC8254125

[151] Zhang J-T Ma S-S Yip SW Wang L-J Chen C Yan C-G Decreased functional connectivity between ventral tegmental area and nucleus accumbens in Internet gaming disorder: evidence from resting state functional magnetic resonance imaging. *Behav Brain Funct.* (2015) 11:37. 10.1186/s12993-015-0082-8 26582309 PMC4652358

[152] Wagner G de la Cruz F Köhler S Bär K-J. Treatment associated changes of functional connectivity of midbrain/brainstem nuclei in major depressive disorder. *Sci Rep.* (2017) 7:8675. 10.1038/s41598-017-09077-5 28819132 PMC5561091

[153] Anand A Jones SE Lowe M Karne H Koirala P. Resting state functional connectivity of dorsal raphe nucleus and ventral tegmental area in medication-free young adults with major depression. *Front Psychiatry.* (2018) 9:765. 10.3389/fpsyt.2018.00765 30761028 PMC6362407

[154] Li W Lao-Kaim NP Roussakis A-A Martín-Bastida A Valle-Guzman N Paul G Longitudinal functional connectivity changes related to dopaminergic decline in Parkinson’s disease. *Neuroimage Clin.* (2020) 28:102409. 10.1016/j.nicl.2020.102409 32916466 PMC7490914

[155] Depierreux F Parmentier E Mackels L Baquero K Degueldre C Balteau E Parkinson’s disease multimodal imaging: F-DOPA PET, neuromelanin-sensitive and quantitative iron-sensitive MRI. *NPJ Parkinsons Dis.* (2021) 7:57. 10.1038/s41531-021-00199-2 34238927 PMC8266835

[156] Cho KIK Shenton ME Kubicki M Jung WH Lee TY Yun J-Y Altered thalamo-cortical white matter connectivity: probabilistic tractography study in clinical-high risk for psychosis and first-episode psychosis. *Schizophr Bull.* (2016) 42:723–31. 10.1093/schbul/sbv169 26598740 PMC4838094

[157] D’Ambrosio E Jauhar S Kim S Veronese M Rogdaki M Pepper F The relationship between grey matter volume and striatal dopamine function in psychosis: a multimodal 18F-DOPA PET and voxel-based morphometry study. *Mol Psychiatry.* (2021) 26:1332–45. 10.1038/s41380-019-0570-6 31690805 PMC7610423

[158] Pauli WM Nili AN Tyszka JM. A high-resolution probabilistic *in vivo* atlas of human subcortical brain nuclei. *Sci Data.* (2018) 5:180063. 10.1038/sdata.2018.63 29664465 PMC5903366

[159] Nakamura Y Okada N Kunimatsu A Kasai K Koike S. Anatomical templates of the midbrain ventral tegmental area and substantia nigra for asian populations. *Front Psychiatry.* (2018) 9:383. 10.3389/fpsyt.2018.00383 30210369 PMC6121162

[160] Teipel SJ Flatz WH Heinsen H Bokde ALW Schoenberg SO Stöckel S Measurement of basal forebrain atrophy in Alzheimer’s disease using MRI. *Brain.* (2005) 128:2626–44. 10.1093/brain/awh589 16014654

[161] Paxinos G Huang X-F. *Atlas of the Human Brainstem.* Amsterdam: Elsevier (2013). 149 p.

[162] Ahsan RL Allom R Gousias IS Habib H Turkheimer FE Free S Volumes, spatial extents and a probabilistic atlas of the human basal ganglia and thalamus. *Neuroimage.* (2007) 38:261–70. 10.1016/j.neuroimage.2007.06.004 17851093

[163] Trutti AC Fontanesi L Mulder MJ Bazin P-L Hommel B Forstmann BU. A probabilistic atlas of the human ventral tegmental area (VTA) based on 7 Tesla MRI data. *Brain Struct Funct.* (2021) 226:1155–67. 10.1007/s00429-021-02231-w 33580320 PMC8036186

[164] Haacke EM Liu S Buch S Zheng W Wu D Ye Y. Quantitative susceptibility mapping: current status and future directions. *Magn Reson Imaging.* (2015) 33:1–25. 10.1016/j.mri.2014.09.004 25267705

[165] Wolfers T Doan NT Kaufmann T Alnæs D Moberget T Agartz I Mapping the heterogeneous phenotype of schizophrenia and bipolar disorder using normative models. *JAMA Psychiatry.* (2018) 75:1146–55. 10.1001/jamapsychiatry.2018.2467 30304337 PMC6248110

[166] Di Biase MA Geaghan MP Reay WR Seidlitz J Weickert CS Pébay A Cell type-specific manifestations of cortical thickness heterogeneity in schizophrenia. *Mol Psychiatry.* (2022) 27:2052–60. 10.1038/s41380-022-01460-7 35145230 PMC9126812

[167] Katsel P Roussos P Pletnikov M Haroutunian V. Microvascular anomaly conditions in psychiatric disease. Schizophrenia - angiogenesis connection. *Neurosci Biobehav Rev.* (2017) 77:327–39. 10.1016/j.neubiorev.2017.04.003 28396239 PMC5497758

[168] Edlow BL. Dopaminergic modulation of human consciousness *via* default mode network connectivity. *Proc Natl Acad Sci USA.* (2021) 118:e2111268118. 10.1073/pnas.2111268118 34330840 PMC8346910

[169] Harris JP Burrell JC Struzyna LA Chen HI Serruya MD Wolf JA Emerging regenerative medicine and tissue engineering strategies for Parkinson’s disease. *npj Parkinsons Dis.* (2020) 6:1–14. 10.1038/s41531-019-0105-5 31934611 PMC6949278

[170] Edlow BL Takahashi E Wu O Benner T Dai G Bu L Neuroanatomic connectivity of the human ascending arousal system critical to consciousness and its disorders. *J Neuropathol Exp Neurol.* (2012) 71:531–46. 10.1097/NEN.0b013e3182588293 22592840 PMC3387430

[171] Bär K-J de la Cruz F Schumann A Koehler S Sauer H Critchley H Functional connectivity and network analysis of midbrain and brainstem nuclei. *Neuroimage.* (2016) 134:53–63. 10.1097/NEN.0b013e3182588293 27046112

[172] Avram M Brandl F Knolle F Cabello J Leucht C Scherr M Aberrant striatal dopamine links topographically with cortico-thalamic dysconnectivity in schizophrenia. *Brain.* (2020) 143:3495–505. 10.1093/brain/awaa296 33155047

[173] Watanabe Y Tanaka H Tsukabe A Kunitomi Y Nishizawa M Hashimoto R Neuromelanin magnetic resonance imaging reveals increased dopaminergic neuron activity in the substantia nigra of patients with schizophrenia. *PLoS One.* (2014) 9:e104619. 10.1371/journal.pone.0104619 25111500 PMC4128756

